# The evolution of Jen3 proteins and their role in dicarboxylic acid transport in *Yarrowia*

**DOI:** 10.1002/mbo3.225

**Published:** 2014-12-16

**Authors:** Rémi Dulermo, Heber Gamboa-Meléndez, Stéphanie Michely, France Thevenieau, Cécile Neuvéglise, Jean-Marc Nicaud

**Affiliations:** 1 UMR1319 Micalis, INRAJouy-en-Josas, F-78352, France; 2 UMR Micalis, AgroParisTechJouy-en-Josas, F-78352, France; 3 Direction Innovation, SOFIPROTEOLParis, F-75378, France

**Keywords:** Fumarate, lactate, malate, succinate, transport, yeast

## Abstract

Jen proteins in yeast are involved in the uptake of mono/dicarboxylic acids. The Jen1 subfamily transports lactate and pyruvate, while the Jen2 subfamily transports fumarate, malate, and succinate. *Yarrowia lipolytica* has six *JEN* genes: *YALI0B19470g*, *YALI0C15488g*, *YALI0C21406g*, *YALI0D20108g*, *YALI0D24607g*, and *YALI0E32901g*. Through phylogenetic analyses, we found that these genes represent a new subfamily, Jen3 and that these three Jen subfamilies derivate from three putative ancestral genes. Reverse transcription-PCR. revealed that only four *YLJEN* genes are expressed and they are upregulated in the presence of lactate, pyruvate, fumarate, malate, and/or succinate, suggesting that they are able to transport these substrates. Analysis of deletion mutant strains revealed that Jen3 subfamily proteins transport fumarate, malate, and succinate. We found evidence that *YALI0C15488* encodes the main transporter because its deletion was sufficient to strongly reduce or suppress growth in media containing fumarate, malate, or succinate. It appears that the other *YLJEN* genes play a minor role, with the exception of *YALI0E32901g*, which is important for malate uptake. However, the overexpression of each *YLJEN* gene in the sextuple-deletion mutant strain Δ*YLjen1-6* revealed that all six genes are functional and have evolved to transport different substrates with varying degrees of efficacy. In addition, we found that YALI0E32901p transported succinate more efficiently in the presence of lactate or fumarate.

## Introduction

Jen proteins are transporters of mono- and dicarboxylic acids, which are localized in the plasma membrane (Paiva et al. 2002; Vieira et al. 2010). Their functions were first described in *Saccharomyces cerevisiae* (Casal et al. 1999), in which the expression and activity of SCJen1p (encoded by *YKL217W*) are (1) regulated through a complex pathway which involves different stages of expression (transcriptional, translational, and posttranslational); (2) strain-dependent; and (3) strongly affected by the carbon source present, especially in the case of glucose (Andrade and Casal 2001; Paiva et al. 2002; Andrade et al. 2005). Interestingly, glucose, fructose, sucrose, and mannose have been shown to inhibit the expression of *SCJEN1*, whereas galactose, raffinose, ethanol, glycerol, and lactate have the opposite effect (Andrade and Casal 2001; Paiva et al. 2002; Tsuboi et al. 2003; Chambers et al. 2004). SCJen1p has been shown to be responsible for the active transport of lactate and pyruvate in *S. cerevisiae* (Casal et al. 1999; Akita et al. 2000), while in *Candida albicans*, CAJen1p can actively transport lactate, pyruvate, and propionate (Soares-Silva et al. 2004). Moreover, a recent study showed that SCJen1p is involved in selenite transport (McDermott et al. 2010).

Some species, like *C. albicans* and *Kluyveromyces lactis*, have two types of Jen proteins. In addition to *JEN1*, they also possess a *JEN2* gene, which is repressed in the presence of glucose and upregulated when exposed to succinate or malate (Lodi et al. 2004; Vieira et al. 2010). As mentioned above, CAJen1p transports lactate, pyruvate, and propionate (Soares-Silva et al. 2004), while Jen2p is a fumarate, succinate, and malate transporter in both *C. albicans* and *K. lactis* (Lodi et al. 2004; Queirós et al. 2007; Vieira et al. 2010). In species with two types of Jenp, the disruption of both *JEN1* and *JEN2* affects growth on mono- and dicarboxylic acids (Lodi et al. 2004; Vieira et al. 2010). For example, a Δ*CAjen1*Δ*CAjen2* mutant displayed a growth defect on single-carbon-source media containing lactic, succinic, malic, or pyruvic acids (Vieira et al. 2010).

The genome of *Yarrowia lipolytica* encodes six proteins that are homologous to Jen proteins: YALI0B19470p (YLJen5p), YALI0C15488p (YLJen1p), YALI0C21406p (YL-Jen2p), YALI0D20108p (YLJen3p), YALI0D24607p (YLJen4p), and YALI0E32901p (YLJen6p). Phylogenetic analyses by Lodi et al. (2007) revealed that Jen proteins in *Y. lipolytica* form a group separate from those in *S. cerevisiae*, *K. lactis*, *C. albicans*, and other Hemiascomycetes and Euascomycetes. In the same study, a comparison of gene synteny assigned *YLJEN2*, *YLJEN3*, *YLJEN4*, and *YLJEN6* to the Jen2 cluster, and *YLJEN5* and *YLJEN1* to the Jen1 cluster (Lodi et al. 2007). The authors also identified three molecular motifs that distinguished Jen2 proteins from Jen1 proteins: the absence of a C-terminal helix, TM11, usually found in Jen1 proteins; a C-terminal proline, rather than histidine, residue; and a sequence gap between transmembrane domains (TMs) 6 and 7 (Lodi et al. 2007). However, they also noted that YLJen1p and YLJen5p did not present the motifs typical of the Jen1 subfamily, and thus classified these two proteins in a “preJen1” cluster. They hypothesized that *preJEN1* represented an intermediate evolutionary step between *JEN2* (the ancestral gene) and *JEN1*.

**Figure 1:**
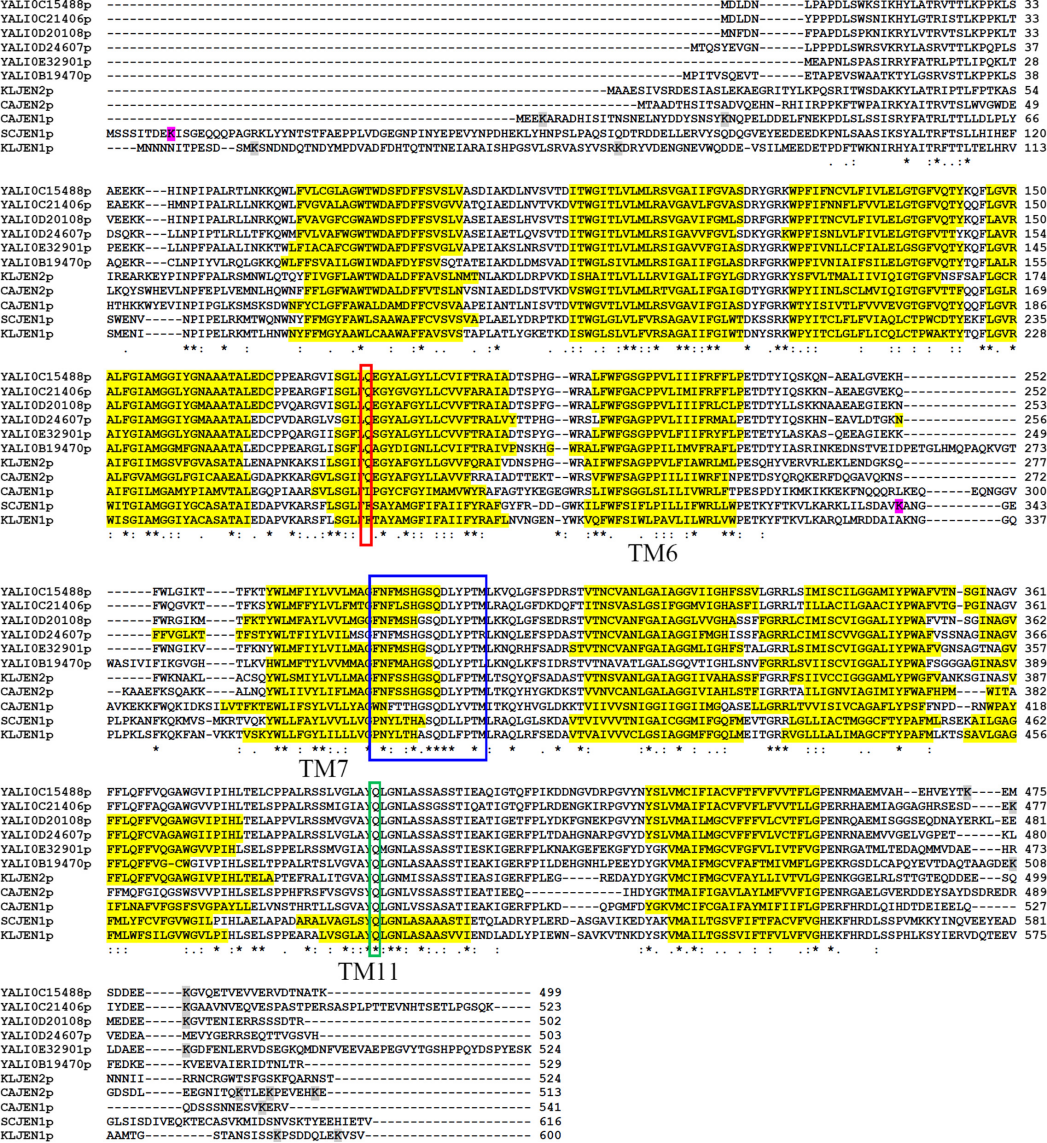
Alignment of the six YLJen protein sequences with Jen1p and/or Jen2p sequences of *Saccharomyces cerevisiae*, *Kluyveromyces lactis*, and *Candida albicans*, constructed with ClustalW. SC, *S. cerevisiae* (YKL217W for SCJen1p), CA, *C. albicans* (CaO19.7447 for CAJen1p; CaO19.12767 for CAJen2p) and KL, *K. lactis* (KLLA0E16259 for KLJen1p; KLLA0F10043 for KLJen2p). The blue frame contains the NXX(S/T)HX(S/T)QDXXXT motif and the F270 and Q498 residues of *S. cerevisiae* are indicated by red and green frames, respectively. Yellow-highlighted amino acids represent transmembrane domains predicted by TMHMM. Gray-highlighted amino acids represent putative ubiquitination sites predicted with high confidence by www.ubpred.org. Purple-highlighted amino acids indicate K9 and K338, the experimentally confirmed ubiquitination sites of SCJen1p (Paiva et al. 2009).

All six Jen proteins in *Y. lipolytica* share a conserved motif in the seventh TM, **N**XX(S/T)**H**X(S/T)**QD**XXX**T**, which is involved in transport ability and substrate affinity (Soares-Silva et al. 2007; Fig.1). Indeed, substitution at the N379, H383, or D387 residues of SCJen1p resulted in a major reduction in lactate and pyruvate uptake, but did not affect acetate transport. Instead, mutations at the Q386 and T391 residues resulted in no or only moderate changes in Jen1p's ability to transport lactate, pyruvate, and acetate, but did result in modifications in its respective affinity for each acid. The same study also presented evidence for the existence of a charged interaction between amino acids H383 and D387 which contributes to Jen1p's protein structure. Interestingly, lactate and pyruvate transport were completely suppressed in cells with the double mutation H383D/D387H, while the kinetics of acetate transport were similar to those found with wild-type Jen1p (Soares-Silva et al. 2007).

As the synteny analysis of Lodi et al. (2007) was poorly supported and that the phylogeny placed the six *Y. lipolytica* genes in a monophyletic group, we investigated the possibility that these genes derive from a species-specific gene expansion event. Such an expansion could have conferred unique characteristics on the Jen proteins in *Y. lipolytica* that would distinguish them from the Jen proteins in other species, we also explored the substrate specificity of each protein. Through comparative genomics analyses involving species closely related to *Y. lipolytica,* we found that this protein family has a dynamic evolutionary history dating from the emergence of the *Yarrowia* clade and constitutes a new subfamily of Jen proteins, Jen3. To analyze the specificity and function of each YLJenp, we first constructed multiple mutant strains and then expressed each *YLJEN* gene in a Δ*YLjen1-6* background. We found that the most important proteins, that is, the ones whose absence had the largest effects on cell function, were YLJen1p and YLJen6p. We also show that Jen3p proteins are fumarate, malate, and succinate transporters.

## Material and Methods

### Yeast growth and culture conditions

The *Y. lipolytica* strains used in this study were derived from the wild-type *Y. lipolytica* W29 (ATCC20460) strain (Table1). The auxotrophic strain Po1d (Leu^−^ Ura^−^) has been previously described by Barth and Gaillardin (1996). The prototrophic strain JMY2900 was obtained by transformation of JMY330, a po1d Ura^+^ derivative (Beopoulos et al. 2008), with a fragment carrying the *LEU2* gene and verification that the strain have recovered the wild-type *LEU2* locus as described by Dulermo et al. (2013). All of the strains used in this study are listed in Table1. Media and growth conditions for *Escherichia coli* have been previously described by Sambrook et al. (1989), and those for *Y. lipolytica* have been described by Barth and Gaillardin (1996). Rich medium (Yeast extract, Peptone, Dextrose (YPD)) and minimal glucose medium (YNB) were prepared as described previously (Mlícková et al. 2004). Minimal medium (Yeast Nitrogen Base (YNB)) contained 0.17% (wt/vol) yeast nitrogen base (without amino acids and ammonium sulfate, YNBww; Difco, Paris, France), 0.5% (wt/vol) NH_4_Cl, and 50 mmol/L phosphate buffer (pH 6.8). During the experiment, this minimal medium was supplemented with uracil (0.1 g/L) and/or leucine (0.1 g/L) if necessary. To test growth on different carbon sources, we used the following medium: 0.17% (wt/vol) yeast nitrogen base (without amino acids and ammonium sulfate, YNBww; Difco), 0.5% (wt/vol) NH4Cl, (Difco), and 50 mmol/L phosphate buffer (pH 6.8). For growth and consumption tests, a carbon source was added at 0.15–0.3% (wt/vol). Solid media were obtained by the addition of 1.6% agar. *Y. lipolytica* precultures were grown in minimal medium to avoid stress on strains that would be used in phenotype tests.

**Table 1:** Strains and plasmids

Strain or plasmid	Genotype or other relevant characteristics (name in figures)	Source or reference
*Escherichia coli*		
DH5*α*	*Φ80dlacZΔm15, recA1, endA1, gyrA96, thi-1, hsdR17 (r*_*k*_*−, m*_*k*_*+), supE44, relA1, deoR, Δ(lacZYA-argF)U169*	Promega
*Yarrowia lipolytica*		
W29	*MatA, wild-type*	Barth and Gaillardin (1996)
Po1d	*MatA ura3*-302 *leu2*-270 *xpr*2-322	Barth and Gaillardin (1996)
JMY330	*MatA ura3*-302 *leu2*-270 *xpr*2-322, *URA3ex;* Ura^+^ Leu^−^	Beopoulos et al. (2008)
JMY2900	*MatA ura3*-302 *xpr*2-322, *URA3ex;* Ura^+^ Leu^+^	F. Brunel, (unpubl. data)
JMY2946	Po1d Δ*YALI0B19470*::*URA3ex* Leu^−^	This study
JMY2949	Po1d *YALI0C21406*::*URA3ex YALI0C15488*::*LEU2ex* (Δ*YLjen1,2*)	This study
JMY2956	Po1d Δ*YALI0C15488*::*URA3ex* Leu^−^	This study
JMY3007	Po1d Δ*YALI0C21406*::*URA3ex* Leu^−^	This study
JMY3012	Po1d Δ*YALI0D20108*::*URA3ex* Leu^−^	This study
JMY3014	Po1d *YALI0C15488*::*URA3ex YALI0B19470*::*LEU2ex* (Δ*YLjen1,5*)	This study
JMY3018	Po1d *YALI0C21406*::*URA3ex YALI0B19470*::*LEU2ex* (Δ*YLjen2,5*)	This study
JMY3040	Po1d *YALI0E32901*::*URA3ex* Leu^−^	This study
JMY3075	Po1d *YALI0D20108*::*URA3ex YALI0B19470*::*LEU2ex* (Δ*YLjen3,5*)	This study
JMY3084	Po1d Δ*YALI0*C21406 Δ*YALI0*C15488 Ura^−^ Leu^−^	This study
JMY3086	Po1d Δ*YALI0*C15488 Δ*YALI0*B19470 Ura^−^ Leu^−^	This study
JMY3088	Po1d Δ*YALI0*C21406 Δ*YALI0*B19470 Ura^−^ Leu^−^	This study
JMY3090	Po1d *YALI0B19470*::*URA3ex YALI0E32901*::*LEU2ex* (Δ*YLjen5,6*)	This study
JMY3092	Po1d *YALI0C15488*::*URA3ex YALI0E32901*::*LEU2ex* (Δ*YLjen1,6*)	This study
JMY3093	Po1d *YALI0D20108*::*URA3ex YALI0E32901*::*LEU2ex* (Δ*YLjen3,6*)	This study
JMY3094	Po1d *YALI0D20108*::*URA3ex YALI0C15488*::*LEU2ex* (Δ*YLjen1,3*)	This study
JMY3101	Po1d *YALI0B19470*::*URA3ex* + *LEU2ex* (Δ*YLjen5*)	This study
JMY3104	Po1d *YALI0C15488*::*URA3ex* + *LEU2ex* (Δ*YLjen1*)	This study
JMY3106	Po1d *YALI0C21406*::*URA3ex* + *LEU2ex* (Δ*YLjen2*)	This study
JMY3109	Po1d *YALI0D20108*::*URA3ex* + *LEU2ex* (Δ*YLjen3*)	This study
JMY3112	Po1d *YALI0E32901*::*URA3ex* + *LEU2ex* (Δ*YLjen6*)	This study
JMY3146	JMY3084 + *YALI0D20108*::*URA3ex* Leu^−^	This study
JMY3147	JMY3086 + *YALI0D20108*::*URA3ex* Leu^−^	This study
JMY3151	JMY3084 + *YALI0B19470*::*URA3ex* Leu^−^	This study
JMY3152	JMY3084 + *YALI0B19470*::*URA3ex* Leu^−^	This study
JMY3156	JMY3088 + *YALI0D20108*::*URA3ex* Leu^−^	This study
JMY3171	JMY3147 + *YALI0E32901*::*LEU2ex* (Δ*YLjen1,3,5,6*)	This study
JMY3209	JMY3151 + *LEU2ex* (ΔYLjen*1,2,5*)	This study
JMY3210	JMY3156 + *LEU2ex* (ΔYLjen*2,3,5*)	This study
JMY3213	JMY3156 + *YALI0E32901*::*LEU2ex* (Δ*YLjen2,3,5,6*)	This study
JMY3215	JMY3171 Ura^−^ Leu^−^	This study
JMY3249	JMY3156 + *YALI0D24607*::*LEU2ex* (Δ*YLjen2,3,4,5*)	This study
JMY3250	JMY3151 + *YALI0E32901*::*LEU2ex* (Δ*YLjen1,2,5,6*)	This study
JMY3252	Y3147 + *YALI0D24607*::*LEU2ex* (Δ*YLjen1,3,4,5*)	This study
JMY3257	Po1d + *YALI0D24607*::*LEU2ex* Ura^−^	This study
JMY3278	JMY3257 + *URA3ex* (ΔYLjen*4*)	This study
JMY3282	JMY3215 + *YALI0D24607*::*LEU2ex* Ura^−^	This study
JMY3333	JMY3282 + *URA3ex* (Δ*YLjen1,3,4,5,6*)	This study
JMY3598	JMY3282 + *YALI0C21406*::*URA3ex* (Δ*YLjen1-6*)	This study
JMY3702	JMY3598 Ura^−^ Leu^−^	This study
JMY3722	JMY3598 *LEU2ex* Ura^−^	This study
JMY3751	JMY3722 + p*TEF*-*YALI0B19470*-*URA3ex* (*YLJEN5*^++^)	This study
JMY3754	JMY3722 + p*TEF*-*YALI0C15488*-*URA3ex* (*YLJEN1*^++^)	This study
JMY3757	JMY3722 + p*TEF*-*YALI0D20108*-*URA3ex* (*YLJEN3*^++^)	This study
JMY3760	JMY3722 + p*TEF*-*YALI0D24607*-*URA3ex* (*YLJEN4*^++^)	This study
JMY3763	JMY3722 + p*TEF*-*YALI0E32901*-*URA3ex* (*YLJEN6*^++^)	This study
JMY4125	JMY3702 + p*TEF*-*C21406*-*LEU2ex* Ura^−^	This study
JMY4157	JMY4125 + *URA3ex* (*YLJEN2*^++^)	This study
Plasmids		
pCR4Blunt-TOPO	Cloning vector	Invitrogen
JMP802	JMP62-pTEF-*LEU2*ex	
JMP803	JMP62-pTEF-*URA3ex*	(Müller et al. 1998; Nicaud et al. 2002)
JMP547	pUB4-CRE	Fickers et al. (2003)
JMP1392	JMP62-p*TEF*-*RedStar2SKL*-*LEU2ex*	Kabran et al. (2012)
JMP1647	pCR4Blunt-TOPO *YALI0B19470*UpDn	This study
JMP1649	pCR4Blunt-TOPO *YALI0C15488*UpDn	This study
JMP1683	pCR4Blunt-TOPO *YALI0C21406*UpDn	This study
JMP1684	pCR4Blunt-TOPO *YALI0D20108*UpDn	This study
JMP1689	pCR4Blunt-TOPO *YALI0E32901*UpDn	This study
JMP1667	pCR4Blunt-TOPO *YALI0B19470*UpDn-*URA3ex*	This study
JMP1671	pCR4Blunt-TOPO *YALI0C15488*UpDn-*URA3ex*	This study
JMP1679	pCR4Blunt-TOPO *YALI0B19470*UpDn-*LEU2ex*	This study
JMP1681	pCR4Blunt-TOPO *YALI0C15488*UpDn-*LEU2ex*	This study
JMP1698	pCR4Blunt-TOPO *YALI0C21406*UpDn-*URA3ex*	This study
JMP1702	pCR4Blunt-TOPO *YALI0D20108*UpDn-*URA3ex*	This study
JMP1713	pCR4Blunt-TOPO *YALI0E32901*UpDn-*URA3ex*	This study
JMP1715	pCR4Blunt-TOPO *YALI0E32901*UpDn-*LEU2ex*	This study
JMP 1792	JMP62 p*TEF*-*YALI0B19470-URA3ex*	This study
JMP1794	JMP62 p*TEF*-*YALI0C15488-URA3ex*	This study
JMP1796	JMP62 p*TEF*-*YALI0D24607-URA3ex*	This study
JMP1798	JMP62 p*TEF*-*YALI0E32901-URA3ex*	This study
JMP1809	JMP62 p*TEF*-*YALI0D20108-URA3ex*	This study
JMP1813	pCR4Blunt-TOPO *YALI0D24607UpDn*-*LEU2ex*	This study
JMP2366	JMP62 p*TEF*-*YALI0C21406*-*LEU2ex*	This study

### General genetic techniques

Standard molecular genetic techniques were used throughout this study (Sambrook et al. 1989). Restriction enzymes were obtained from OZYME (Saint-Quentin-en-Yvelines, France). Yeast cells were transformed using the lithium acetate technique (Le Dall et al. 1994). Genomic DNA from yeast transformants was prepared as described by Querol et al. (1992). PCR amplifications were performed in an Eppendorf 2720 thermal cycler with GoTaq DNA polymerases (Promega, Charbonniére-les-Bains, France) for verification PCR and PyroBest DNA polymerases (Takara, Saint-Germain-en-laye, France) for cloning. Table2 gives the primers that were used during this study. PCR fragments were purified with the QIAgen Purification Kit (Qiagen, Courtaboeuf, France), and DNA fragments were recovered from agarose gels with the QIAquick Gel Extraction Kit (Qiagen). The Clone Manager software package was used for gene sequence analysis Sci-Ed Software, Scientific and Education Softwara, Morrisville, NC, USA.

**Table 2:** Primer list

Genes	Primers	Sequences	Utilization
*YALI0B19470g* (*YLJEN5*)	B19470UpNotI	GAATGCGGCCGCCAGTTGGTTCAACTGGGTCC	Upstream fragment of *YALI0B19470*
	B19470UpIsceI	CGATTACCCTGTTATCCCTACCGGCATGAGAGTGGTGTTTGG	
	B19470DnNotI	GAATGCGGCCGCCAGCCAGGTCAGCTACTATT	Downstream fragment of *YALI0B19470*
	B19470DnIsceIIceuI	GGTAGGGATAACAGGGTAATCGTAACTATAACGGTCCTAAGGTAGCGACCAATCTCACTCGTTAATTAG	
	Ver1B19470	CAAGTATCATGGTTTGTGGG	Verification of disruption
	Ver2B19470	GTGTCACAACAGTGTCACCC	
	StartB19470	ATCGGATCCCACAATGCCCATCACAGTTTCACAAGAAGTG	Overexpression of *YALI0B19470*
	EndB19470	CATCCTAGGTTAACGAGTGAGATTGGTGTCGATTCG	
	B19470F	TCTTCTTCTTGCAGTTCTTTG	Expression of *YALI0B19470* by RT-PCR
	B19470R	GTGCTCATCGAGAATAGGG	
*YALI0C15488g* (*YLJEN1*)	C15488UpNotI	GAATGCGGCCGCTTTGTCTGTCTTCCCGTTGC	Upstream fragment of *YALI0C15488*
	C15488UpIsceI	CGATTACCCTGTTATCCCTACCGGTGAATGAACGAACGAAGAAGG	
	C15488DnNotI	GAATGCGGCCGCTGAGAGTCCTTCTGTCTACC	Downstream fragment of *YALI0C15488*
	C15488DnIsceIIceuI	GGTAGGGATAACAGGGTAATCGTAACTATAACGGTCCTAAGGTAGCGAGTGTAGCAACTAACGTATTG	
	Ver1-1C15488	CCACTCCTCCTCTCCTTAGACCG	Verification of disruption
	Ver2-2C15488	CCTCTCCATCGATTTCGAGGTGC	
	StartC15488	ATCGGATCCCACAATGGATTTGGACAACCTCCCTGCCCC	Overexpression of *YALI0C15488*
	EndC15488	CATCCTAGGCTACTTAGTAGCATTGGTGTCAACTC	
	C15488F	TTCTTCTTGCAGTTCTTCGT	Expression of *YALI0C15488* by RT-PCR
	C15488R	CGACCCCATTATCATCTTT	
*YALI0C21406g* (*YLJEN2*)	C21406UpNotI	GAATGCGGCCGCGGAGAAAATGGACGTGTGAGACGC	Upstream fragment of *YALI0C21406*
	C21406UpIsceI	CGATTACCCTGTTATCCCTACCGAGATAGAGCAAGTAGAAGCAGCG	
	C21406DnNotI	GAATGCGGCCGCGGACTTGTAACAGCACACGTTCGC	Downstream fragment of *YALI0C21406*
	C21406Dn2IsceIIceuI	GGTAGGGATAACAGGGTAATCGTAACTATAACGGTCCTAAGGTAGCGACTCTGAGACCCTCCCCGGATCCC	
	Ver1C21406	CATTAGCGTGGTTTCCATGCG	Verification of disruption
	Ver2C21406	AAGAGAGTTACTTTTGCGGGAGG	
	StartC21406	ATCGGATCCCACAATGGATCTCGACAACTACCCTCCTC	Overexpression of *YALI0C21406*
	EndC21406	CATCCTAGGTCACTTTTGGGATCCGGGGAGGGTCTC	
	C21406F	GATCTTCCATGATTGGTATTG	Expression of *YALI0C21406* by RT-PCR
	C21406R	CAGGGTGACGACAAAGAG	
*YALI0D20108g* (*YLJEN3*)	D20108UpNotI	GAATGCGGCCGCGCACAAGTAGGTGGGTCCTTCCG	Upstream fragment of *YALI0D20108*
	D20108UpIsceI	CGATTACCCTGTTATCCCTACCGACAGATTGAGCGAGTAGACAGG	
	D20108DnNotI	GAATGCGGCCGCGAGAATCTGGAAGCATCCTGCTGCC	Downstream fragment of *YALI0D20108*
	D20108Dn2IsceIIceuI	GGTAGGGATAACAGGGTAATCGTAACTATAACGGTCCTAAGGTAGCGATAATGTAGGGTGTGTGTGTGACG	
	Ver1-2D20108	CCAAGGGTGGCCATTAGGAGTCG	Verification of disruption
	Ver2-2D20108	GGTGTCATATTCGAGTCCTCGC	
	StartD20108-2	ATCGGATCCCACAATGAATTTTGACAACTTCCCAGCACCAGATCTG	Overexpression of *YALI0D20108*
	EndD20108-2	CATCCTAGGTTATCGAGTATCGCTCGAAGAACGTCTTTCAATGTTC	
	D20108F	CTTCTTCTGCTTCTTCAACC	Expression of *YALI0D20108* by RT-PCR
	D20108R	CATCTCTGCTTGTCTGTTTTC	
*YALI0D24607g* (*YLJEN4*)	D24607UpNotI	GAATGCGGCCGCGATGCAGATCTTTCCGAGCGCTGG	Upstream fragment of *YALI0D24607*
	D24607UpIsceI	CGATTACCCTGTTATCCCTACCGGAAGGAGTCTTCCTGTTTATGG	
	D24607DnNotI	GAATGCGGCCGCCAAGTGGTTCCTGGACCGAATGGC	Downstream fragment of *YALI0D24607*
	D24607Dn2IsceIIceuI	GGTAGGGATAACAGGGTAATCGTAACTATAACGGTCCTAAGGTAGCGAGGCAGGTATTAGATTTATATGAGTAGACG	
	Ver1D24607	CGACATTTGAAGGAGATGACGG	Verification of disruption
	Ver2D24607	GGACATTGTGCCCTGGGCCACC	
	StartD24607	ATCGGATCCCACAATGACCCAGTCGTACGAAGTCGGAAAC	Overexpression of *YALI0D24607*
	EndD24607	CATCCTAGGCTAATGAACACTTCCAACAGTGGTCTG	
	HG-RT-D24607F2	CCGGAGGAATCTTTATGG	Expression of *YALI0D24607* by RT-PCR
	HG-RT-D24607R2	GTGAGATGGATGGGGATG	
*YALI0E32901g* (*YLJEN6*)	E32901UpNotI	GAATGCGGCCGCAGAGAGTTCTTTATCCACCCCACGG	Upstream fragment of *YALI0E32901*
	E32901UpIsceI	CGATTACCCTGTTATCCCTACCGGACTAGTGAGTGCTTGCCACGAG	
	E32901DnNotI	GAATGCGGCCGCCAGTACTCATTACCAGGCAATACGG	Downstream fragment of *YALI0E32901*
	E32901Dn2IsceIIceuI	GGTAGGGATAACAGGGTAATCGTAACTATAACGGTCCTAAGGTAGCGACCAATATGACTCCCCCTACGAGTCC	
	Ver1 E32901	CTCAACAAAGAGATGATAAGCC	Verification of disruption
	Ver2 E32901	CTGTATTGAATATTTGACTGCTCC	
	StartE32901	ATCGGATCCCACAATGGAAGCTCCTAATCTCTCGCCAGC	Overexpression of *YALI0E32901*
	EndE32901	CATCCTAGGTTACTTGGACTCGTAGGGGGAGTC	
	E32901F	CGGCTCTCAAGACCTCTAC	Expression of *YALI0E32901* by RT-PCR
	E32901R	GAGCACCTCCAATAACACAA	
	pTEF-Start	GGGTATAAAAGACCACCGTCC	Verification of insertion of overexpression cassettes
	61 Stop	GTAGATAGTTGAGGTAGAAGTTG	
*Actin*	ACT-A1	TCCAGGCCGTCCTCTCCC	Expression of *Actin* by RT-PCR
	ACT-A2	GGCCAGCCATATCGAGTCGCA	

### Construction of the disruptant strains in *Y. lipolytica*

The deletion cassettes were generated by PCR amplification as described by Fickers et al. (2003) using the primer pairs described in Table2. The Up-Dn cassettes, which contained an *IsceI* site, were then inserted into the pCR®4Blunt-TOPO® vector (Invitrogen, Saint-Aubin, France). The auxotrophic marker (either *URA3ex* or *LEU2ex*) was then inserted into the vector *IsceI* site to generate the corresponding JMP (UpDn*URA3ex*/*LEU2ex*) vectors (Table1). Transformants were selected on YNB and Leu or YNB and Ura, respectively. The corresponding ver1 and ver2 primers (Table2) were used to check gene disruption by PCR amplification of the genomic loci.

For *YALI0B19470g* disruption, the primer pairs B19470UpNotI/B19470UpIsceI and B19470DnNotI/B19470DnIsceIIceuI were used (Table2). The Up and Dn regions were purified and used for the PCR fusion. The resulting UpDn fragment was ligated into pCR4Blunt-TOPO, yielding the construct JMP1647. The *URA3ex* and *LEU2ex* markers (from JMP802 and JMP803) were then introduced at the *I-sce*I site, yielding the constructs JMP1667 and JME1680, which respectively contained the *YALI0B19470g::URA3ex* and *YALI0B19470g::LEU2ex* cassettes. The deletion cassettes used in this study were obtained by digestion of these plasmids by NotI.

For *YALI0C15488g* disruption, primer pairs C15488UpNotI/C15488UpIsceI and C15488DnNotI/C15488DnIsceIIceuI were used (Table2). The Up and Dn regions were purified and used for the PCR fusion. The resulting UpDn fragment was ligated into pCR4Blunt-TOPO, yielding the construct JMP1649. The *URA3ex* and *LEU2ex* markers (from JMP802 and JMP803) were then introduced at the *I-sce*I site, yielding the constructs JMP1671 and JME1682, which respectively contained the *YALI0C15488g::URA3ex* and *YALI0C15488g::LEU2ex* cassettes. Deletion cassettes were obtained by digestion of these plasmids by NotI.

For *YALI0C21406g* disruption, primer pairs C21406UpNotI/C21406UpIsceI and C21406DnNotI/C21406Dn2IsceIIceuI were used (Table2). The Up and Dn regions were purified and used for the PCR fusion. The resulting UpDn fragment was ligated into pCR4Blunt-TOPO, yielding the construct JMP1683. The *URA3ex* marker (from JMP803) was then introduced at the *I-sce*I site, yielding the construct JMP1698, which contained the *YALI0C21406g::URA3ex* cassette. Deletion cassettes were obtained by digestion of these plasmids by NotI.

For *YALI0D20108g* disruption, primer pairs D20108UpNotI/D20108UpIsceI and D20108DnNotI/D20108Dn2IsceIIceuI were used (Table2). The Up and Dn regions were purified and used for the PCR fusion. The resulting UpDn fragment was ligated into pCR4Blunt-TOPO, yielding the construct JMP1684. The *URA3ex* marker (from JMP803) was then introduced at the *I-sce*I site, yielding the construct JMP1702, which contained the *YALI0D20108g::URA3ex* cassette. Deletion cassettes were obtained by digestion of these plasmids by NotI.

For *YALI0D24607g* disruption, primer pairs D24607UpNotI/D24607UpIsceI and D24607DnNotI/D24607Dn2IsceIIceuI were used (Table2). The Up and Dn regions were purified and used for the PCR fusion. The resulting UpDn fragment was ligated into pCR4Blunt-TOPO, yielding the construct JMP1777. The *LEU2ex* marker (from JMP802) was then introduced at the *I-sce*I site, yielding the construct JMP1813, which contained the *YALI0D24607g::LEU2ex* cassette. Deletion cassettes were obtained by digestion of these plasmids by NotI.

For *YALI0E32901g* disruption, primer pairs E32901UpNotI/E32901UpIsceI and E32901DnNotI/E32901Dn2IsceIIceuI were used (Table2). The Up and Dn regions were purified and used for the PCR fusion. The resulting UpDn fragment was ligated into pCR4Blunt-TOPO, yielding the construct JMP1689. The *URA3ex* and *LEU2ex* markers (from JMP802 and JMP803) were then introduced at the *I-sce*I site, yielding the constructs JMP1713 and JME1715, which respectively contained the *YALI0E32901g::URA3ex* and *YALI0E32901g::LEU2ex* cassettes. Deletion cassettes were obtained by digestion of these plasmids by NotI.

Complementation of the sextuple mutant with individual *YLJEN1-6* genes was performed using the respective primer pairs StartB19470/EndB19470, StartC15488/EndC15488, StartC21406/EndC21406, StartD20108/EndD20108, StartD24607/EndD24607, and StartE32901/EndE32901. All complementation genes were cloned into pCR4Blunt-TOPO and then digested by AvrII and BamHI. They were then cloned into JMP1392 that had been previously digested by BamHI and AvrII; this generated JMP1792, JMP1794, JMP1821, JMP1809, JMP1796, and JMP1798, respectively.

Construction of deletion strains was controlled by PCR using Ver1 and Ver2 primers (Table2). Strain complementation was verified by PCR using pTEF-Start and 61 Stop primers.

### Growth microtiter plate analysis

*Y. lipolytica* strain precultures were grown overnight, then centrifuged and washed two times with YNB supplemented with NH_4_Cl and phosphate buffer (YNB N and P). They were then resuspended into 1 mL of YNB N and P. Washed cells were then sown into 96-well microplates with adapted medium at OD_600_ = 0.1 in 200 *μ*L of final volume. Growth was observed using a microtiter plate reader (Biotek, Colmar, France), following the manufacturer's instructions.

### Analysis of *YLJEN* gene expression

To determine if carbon sources induced the expression of *YLJEN* genes, precultures of the reference strain JMY2900 were established in liquid YNB supplemented with 1% glucose and 0.5% yeast extract; cultures grew for 15 h at 28°C. Cells were then washed with distilled water and transferred into fresh liquid YNB media supplemented with one of the following: 1% glucose, 0.3% lactate, 0.3% fumarate, 0.3% malate, 0.3% pyruvate, or 0.3% succinate. Cultures were incubated in baffled Erlenmeyer flasks at 28°C and 160 rpm, then harvested at 2 and 6 h postinoculation, frozen in liquid nitrogen, and stored at −80°C. RNA was extracted from cells using the RNeasy Mini kit (Qiagen), and 2 *μ*g were treated with DNAse (Ambion; Life Technologies, Saint-Aubain, France). cDNA was synthesized using the Maxima First Strand cDNA synthesis kit (Thermo Fisher Scientific, Villebon sur Yvette, France). PCR was then performed using the GoTaq DNA polymerase kit (Promega) with specific primers designed by the Primer3 program and listed in Table2. PCR reactions were performed with 1 *μ*L of cDNA.

### HPLC analysis

*Y. lipolytica* strain precultures were grown overnight, then centrifuged and washed two times with YNB supplemented with NH_4_Cl and phosphate buffer (YNB N and P). They were then resuspended in 1 mL of YNB N and P. Cultures were inoculated at OD_600 nm_ = 0.1 in 50 mL of YNB medium supplemented with 50, 100, or 200 mmol/L of phosphate buffer (pH = 6.8) depending of the quantity of substrate (1, 2, or 4) and 170 mmol/L each of fumarate, lactate, malate, and succinate, then incubated at 28°C with agitation (160 rpm). To analyze the consumption of carbon sources, a sample of each culture was centrifuged for 1 min at 13 000 rpm; the resulting supernatant was analyzed with HPLC (UltiMate 3000; Dionex-Thermo Fisher Scientific, UK) using a HyperREZ XP carbohydrate H^+^ 8-*μ*m column (Thermo Fisher Scientific, Villebon sur Yvette, France) coupled to a UV (210 nm) detector. The column was eluted with 0.01 N H_2_SO_4_ at room temperature and a flow rate of 0.6 mL min^−1^. Standard samples were used to determine the quantity of each compound. Prior to HPLC analysis, samples were filtered on 0.45-*μ*m pore-size membranes.

### Identification of Jen proteins in ascomycetous yeasts

Twenty-three species were chosen based on their positions in the phylogenetic tree of hemiascomycetes (see Kurtzman 2011; for reference tree). For each species, genomes were retrieved from the indicated database: *S. cerevisiae* – SGD (http://www.yeastgenome.org/); *C. albicans* – CGD (http://www.candidagenome.org/); *Schizosaccharomyces pombe* – Pombase (http://www.pombase.org/); *Komagataella (Pichia) pastoris* – Gent University (https://bioinformatics.psb.ugent.be/gdb/pichia/); *Wickerhamomyces ciferrii*, *Kazachstania naganishii,* and *Naumovozyma castellii* – the NCBI database (http://www.ncbi.nlm.nih.gov/); *Candida tropicalis* – the Broad Institute (http://www.broadinstitute.org/); *Candida glabrata, Zygosaccharomyces rouxii, Eremothecium (Ashbya) gossypii, K. lactis, Lachancea kluyveri, Lachancea thermotolerans,* and *Debaryomyces hansenii* – Génolevures (http://www.genolevures.org/); *Blastobotrys (Arxula) adeninivorans, Cyberlindnera fabianii,* and *Y. lipolytica* – GRYC (http://gryc.inra.fr). Species closely related to *Y. lipolytica*, that is, *Y. yakushimensis* CBS10253, *Yarrowia galli* CBS9722, *Y. phangngensis* CBS10407, *Y. alimentaria* CBS10151, and *C. hispaniensis* CBS9996, were sequenced and annotated in our laboratory at INRA Thiverval-Grignon (data not shown). Homologues of *YLJEN* genes were found via a homology search based on a two-step reciprocal approach using BLASTp (Altschul et al. 1990). In the first step, each YLJen protein was used as a query for a BLASTp search of the other species, with a cutoff E-value of 1e-10. Then, we used the top hit of each search to perform a reciprocal BLASTp search; if the top hit returned a *Y. lipolytica* gene different from a *JEN* gene, it was discarded. Amino acid sequences of all Jen proteins are given in Table S1. Pairwise comparisons of both amino acid identity and similarity were calculated using aligncopypair (EMBOSS; Rice et al. 2000).

### Jen gene phylogeny

The *JEN* gene tree was constructed based on an alignment of 61 Jen proteins, representing 18 yeast species. The alignment was performed with MultAlin (Corpet 1988) and manually corrected with GeneDoc v2.7.0 (Nicholas et al. 1997). The final 405-aa alignment was used to reconstruct trees with both Neighbor-Joining and Maximum-Likelihood algorithms, with three different amino acid substitution matrices (WAG, Dayhoff, and JTT). The models were corrected by a Γ-law distribution with four categories of evolution rates; both invariable sites and the *α*-parameter of the Γ-law distribution were optimized according to the data. Seaview v4.3.3 was used for these analyses (Gouy et al. 2010).

### Reconstruction of an evolutionary scenario

To reconstruct the evolutionary scenario of the Jen protein family in the *Yarrowia* clade, a species tree based on the concatenation of 912 proteins (data not shown) and a Jenp tree built as described above were reconciled with synteny data. A parsimonious approach was used to minimize the number of duplications and losses of *JEN* genes. For the phylogeny of *JEN* genes within the *Yarrowia* clade, KLJen2p was used as an outgroup. Protein sequences were aligned with MAFFT (Katoh et al. 2002) and edited with Gblocks (Castresana 2000). The phylogenetic tree constructed from the Jen protein alignment was constructed with Seaview v4.3.3 using the BioNJ distance method (Gouy et al. 2010). Bootstrap values were calculated with 100 replicates.

## Results and Discussion

### The six *Y. lipolytica* Jen1 proteins are more similar to members of the Jen2 subfamily

The six YLJen proteins were much more similar to each other (60–78% shared identity; Table3) than they were to Jen proteins from other species (37–47% identity with Jen1p, 44–51% identity with Jen2p). Additionally, all YLJen proteins presented 10 TM domains (Fig. S1). Among the six YLJenp, YALI0B19470p (YLJen5p) seemed to be the most divergent, as it shared the smallest percentage of identity both with the other YLJenp and with sequences from other species, and it presented an additional, unique sequence between TM6 and TM7 at residues 258–279 (PETGLHMQPAQKVGTWASIVI; Fig.1). The six YLJenp were shorter than SCJen1p and KLJen1p, both of which have an N-terminal extension that the YLJenp lacked. The alignment of the six YLJenp sequences with those of Jen1p and/or Jen2p from *S. cerevisiae*, *K. lactis*, and *C. albicans* was consistent with the results of Lodi et al. (2007) in revealing a conserved motif in TM7, **N**XX(S/T)**H**X(S/T)**QD**XXX**T**. One exception to this was found, however, in YALI0B19470p, in which the first S/T residue was replaced by an alanine (Fig.1, blue frame). In *S. cerevisiae*, F270 and Q498 residues are highly important for the determination of mono- or dicarboxylate transport function (Soares-Silva et al. 2011). Here, we found that the Q498 residue of SCJen1p was perfectly conserved in all Jenp (Fig.1, green frame), but this was not the case for F270, which was conserved only in KLJen1p. Instead, in all six YLJenp this phenylalanine was replaced by glutamine, as found in Jen2p of *K. lactis* and *C. albicans* (Fig.1, red frame). This observation suggests that the molecules transported by the YLJenp transporters may be different from those of the Jen1p of *S. cerevisiae* or *K. lactis*, and may potentially be more similar to the Jen2p in *K. lactis* and *C. albicans* in their target specificity (i.e., dicarboxylic acids). We also note that the F270 residue was replaced by a leucine in CAJen1p, but as both F and L are nonpolar amino acids, this change is unlikely to have serious consequences for protein specificity.

**Table 3:**
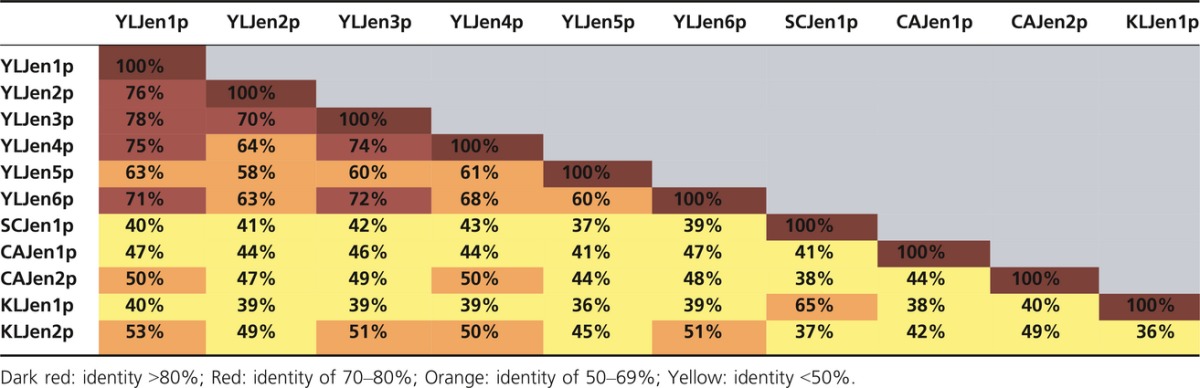
Percentage of shared amino acids between YLJenp and the Jen1p and Jen2p of *Saccharomyces cerevisiae*, *Candida albicans*, and *Kluyveromyces lactis*

SCJen1p has two ubiquitination sites, K9 and K338, but only the latter is involved in the degradation of the protein (Paiva et al. 2009). Whereas all Jen1p had at least one predicted ubiquitination site in the N-terminal domain (K9/K338 for SCJen1p, K4/K28/K538 for CAJen1p, and K14/K589/K597 for KLJen1p), the potential ubiquitination sites of YLJenp and CAJen2p were generally predicted to be in the proteins C-terminal domains: K508 for YALI0B19470p, K473/K481 for YALI0C15488p, K477/K483 for YALI0C21406p, K487 for YALI0D20108p, K479 for YALI0E32901p, and K502/K506 for CAJen2p (Fig.1, highlighted in gray). No ubiquitination site was predicted with high confidence for YALI0D24607p and KLJen2p.

Together, these three results – the observation that YLJenp sequences shared a higher degree of identity with Jen2 proteins than with Jen1p; the replacement of the F240 residue, found in SCJen1p, with a glutamine, as found in the Jen2p sequences; and the difference between YLJenp and Jen1p sequences in the distribution of ubiquitination sites – suggests that the YLJenp are more closely related to the Jen2 protein family than to the Jen1 protein family, in contrast with what has been previously reported by Lodi et al. (2007). To test this hypothesis, we investigated the evolution of these genes by reconstructing the evolutionary history of the Jen family in the *Yarrowia* clade as well as in a broader group of hemiascomycetes.

### Reconstruction of the evolutionary scenario of the JEN family in hemiascomycetes

**Figure 2:**
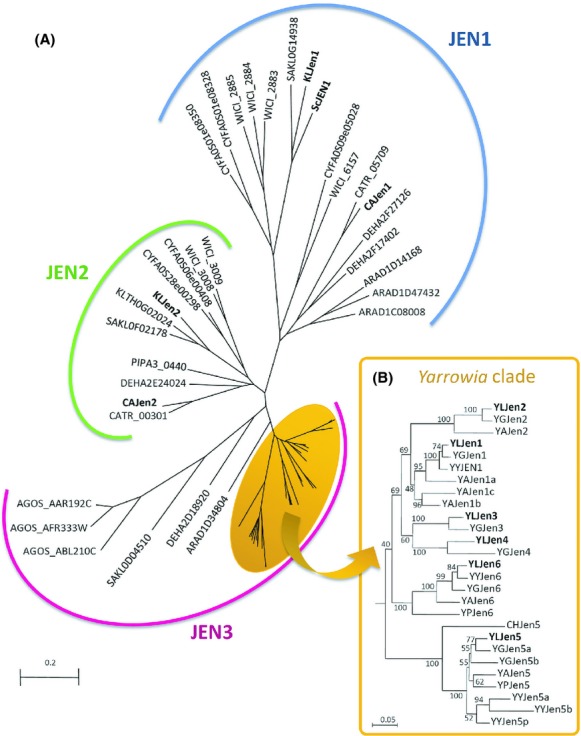
Phylogenetic trees of the Jen protein family. Trees are based on an alignment of Jen proteins and reconstructed with the Neighbor-Joining algorithm. A. Phylogeny of 61 Jen proteins from 18 hemiascomycetous species as inferred from 405 amino acid residues. Each subfamily of Jen proteins is surrounded by a different color: blue for Jen1p, green for Jen2p, and pink for Jen3p. The orange oval indicates the 28 Jen proteins found in the *Yarrowia* clade. Protein names in bold refer to protein from which experimental data have been published. B. Detailed phylogeny of 28 Jen proteins from the *Yarrowia* clade as inferred from an alignment of 423 amino acid residues. KLJen2p was used as an outgroup.

To better understand the evolution of *JEN* genes, we chose 23 species based on their phylogenetic position on the hemiascomycete tree, with the goal of including a broad representation of the genetic diversity within this taxonomic group. We selected nine species of Saccharomycetaceae (four that emerged after the whole genome duplication event (“duplicated”) and five that emerged prior), two species of Wickerhamomycetaceae, and three CTG species, in addition to *Komagataella (Pichia) pastoris, Blastobotrys (Arxula) adeninivorans*, six species closely related to *Y. lipolytica*, and *Schizosaccharomyces pombe,* which served as an outgroup species (Material and Methods). We then retrieved the genomes of these yeasts from genetic databases and attempted to identify the *JEN* genes by BLAST searches; this effort was successful in 18 of the 23 species. With the exception of *S. cerevisiae*, none of the species that diverged after the whole genome duplication event has a *JEN* gene, nor *S. pombe* and *Zygosaccharomyces rouxii*. In total, 61 genes representing 18 species were included in the phylogenetic analysis (Fig.2A). The tree generated from the protein sequences contained three monophylogenetic groups, and the branching patterns within these groups were consistent with the species tree topology (see Kurtzman 2011 for reference tree). Each group corresponded to one of three putative ancestral genes, which then underwent numerous further evolutionary events. This hypothesis is supported by the fact that some species, such as *D. hansenii* and *L. kluyveri*, had genes in each group. The first group included the known genes *SCJEN1*, *KLJEN1*, and *CAJEN1*, as well as genes from *B. adeninivorans*, the CTG, and the Wickerhamomycetaceae. It appears that at least seven duplications occurred in this group, with the most ancestral predating the emergence of the Wickerhamomycetaceae genus and the six others being species-specific duplications. The *KLJEN2* and *CAJEN2* genes belonged to the second monophyletic group, which also contained members of the CTG, the Saccharomycetaceae, the Wickerhamomycetaceae, and *K. pastoris*. The proteins in this group were highly conserved, with pairwise amino acid similarity ranging from 61% to 89%. The branching pattern of the tree indicated that the *JEN2* ancestral gene has been lost in *S. cerevisiae* and, in contrast to our initial hypothesis, none of the *YLJEN* genes clustered with *JEN2* homologues. All branches of the hemiascomycete tree were represented in the third group, which may derive from an ancestral gene that we called *JEN3*. All genes from *Y. lipolytica*, as well as their homologues in closely related species, clustered together within this group, indicating a monophyletic origin. A detailed analysis of their evolutionary history, based on sequence similarity and synteny conservation, revealed an ancestral duplication event which occurred prior to the speciation of *Candida hispaniensis*, a species closely related to *Y. lipolytica*, at the base of the *Yarrowia* clade (Kurtzman 2005; Michely et al. 2013). This duplication led to the evolution of a highly conserved group which contains *YLJEN5*, and a more dynamically evolving group, whose ancestral member has been lost in *C. hispaniensis* (Fig.2B). Three successive duplications occurred in this latter group after the divergence of *C. hispaniensis* and led to the creation of four subgroups. The subgroup represented by *YLJEN6* is highly conserved without any subsequent duplications or losses. In contrast, numerous events (duplications and losses) occurred in the three remaining subgroups. In the group containing the *YLJEN3* and *YLJEN4* genes*,* an additional duplication occurred, but this duplication could not be dated, as all other species, with the exception of *Y. galli*, have lost these two genes. In total, 12 duplications and losses were predicted in the *Yarrowia* clade, indicating that the Jen3 family has a highly dynamic history.

### Transcription of *YLJEN* is substrate-specific and regulated by mono- and dicarboxylic acids

**Figure 3:**
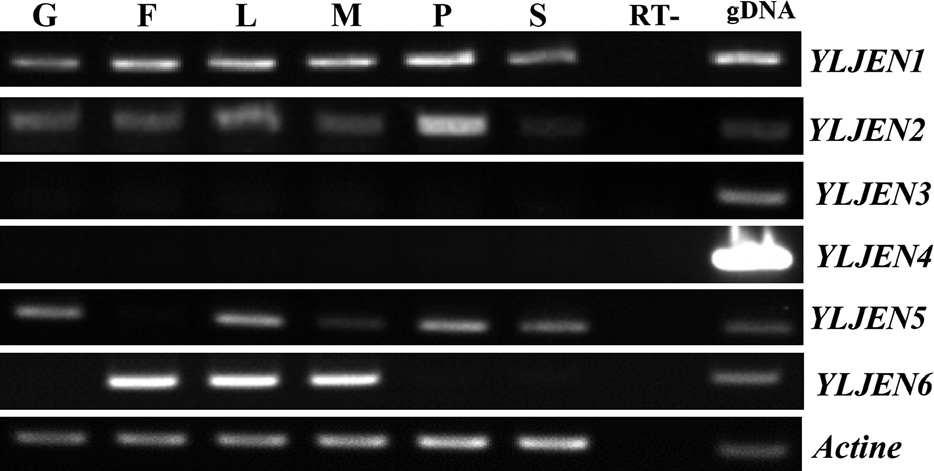
Expression profiles of *YLJEN* genes of reference strain JMY2900 in the presence of glucose, fumarate, lactate, malate, pyruvate, or succinate. Strain precultures were grown in liquid YNB supplemented with 1% glucose and 0.5% yeast extract for 15 h at 28°C (T0) and then transferred into fresh liquid YNB media supplemented with 1% glucose (G), 0.3% fumarate (F), 0.3% lactate (L), 0.3% malate (M), 0.3% pyruvate (P), or 0.3% succinate (S). RT-PCR was performed from cells incubated after 2 h post-inoculation. *Actin* was used as an endogenous control for all conditions tested. RT- and gDNA represent negative and positive controls, respectively.

*SCJEN1* is tightly regulated by various carbon sources; for example, it is repressed by glucose but activated by lactate (Chambers et al. 2004). *KLJEN2* and *CAJEN2* are likewise repressed by glucose and upregulated by succinate and malate (Lodi et al. 2004; Vieira et al. 2010). To determine how *JEN* genes are regulated in *Y. lipolytica*, RT-PCR was performed in media which contained one of the following carbon sources: glucose, fumarate, lactate, malate, pyruvate, and succinate (Fig.3). Surprisingly, the six *YLJEN* genes differed dramatically in their respective expression profiles. *YLJEN1* and *YLJEN2* were expressed under all tested conditions (Fig.3), with increased expression in the presence of lactate and pyruvate. *YLJEN1* was also upregulated in fumarate and malate media, and slightly upregulated in the presence of succinate (Fig.3). *YLJEN1* and *YLJEN2* are closely related in the phylogenetic tree (Fig.2B), suggesting that their common ancestor was also expressed under similar conditions. It is possible that, following the divergence of the two genes, either the promoter of *YLJEN1* evolved so that it became induced by fumarate, malate, and succinate, or, alternatively, this represented an ancestral state, which the promoter of *YLJEN2* subsequently lost.

*YLJEN3* and *YLJEN4* were not expressed in any medium. One potential explanation for this could be that these genes have lost, or are in the process of changing, their ability to be regulated (Fig.3). Interestingly, these genes derivate from the same ancestor (Fig.2B), suggesting that the lack of expression may derive from a common ancestor. Because the lineages *YLJEN1*/*YLJEN2* and *YLJEN3*/*YLJEN4* share a common ancestor, it is tempting to think that the promoter of the *YLJEN3/YLJEN4* ancestor was lost during the duplication event that created these two lineages, which would explain the absence of expression of both modern genes. However, we cannot exclude the hypotheses that *YLJEN3* and *YLJEN4* are in fact expressed, but too weakly to be detected by RT-PCR, or that their promoters are activated under conditions other than those tested here. Alternatively, these two genes may be in the process of becoming pseudogenes, similar to *YAGA0D19768g* in *Y. galli* (a close relative of *YLJEN4*), a pseudogene that has been lost in all other species.

*YLJEN5* was slightly upregulated in the presence of lactate and pyruvate, repressed by fumarate and, to a lesser extent, malate, and expressed in succinate as in glucose (Fig.3). *YLJEN6* was only expressed when exposed to fumarate, lactate, and malate (Fig.3). In contrast to what has been reported for *SCJEN1*, *KLJEN2*, and *CAJEN2*, glucose did not repress the expression of *YLJEN1*, *YLJEN2*, *YLJEN5*, or *YLJEN6*. In this regard the *YLJEN* genes are similar to other genes involved in the metabolic pathways of *Y. lipolytica*, such as *PEX11* or *FAT1* (Dulermo et al. 2014 and unpublished data), a pattern which suggests that glucose catabolite repression does not occur in *Y. lipolytica*.

These results indicate that the regulation of individual *YLJEN* genes differs depending on the carbon source present but, as for *SCJEN1*, most are induced by lactate. The *YLJEN* gene family has thus probably evolved to enable the uptake of several carbon sources under different environmental conditions. In accordance with the expression patterns generated via RT-PCR, we hypothesized that YLJen1p and YLJen2p are involved in the transport of fumarate, lactate, malate, pyruvate and succinate, while YLJen5p transports lactate, pyruvate and probably succinate, and YLJen6p transports fumarate, lactate, and malate.

### YLJen1p is involved in fumarate, malate, and succinate transport

**Figure 4:**
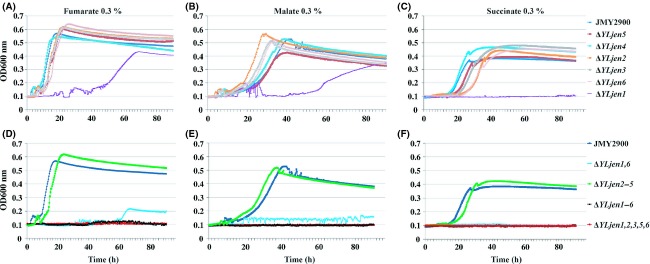
Growth of various *YLJEN*-deletion strains in media which contained 0.3% fumarate, malate, or succinate. (A and D) Growth in fumarate medium. (B and E) Growth in malate medium. (C and F) Growth in succinate medium. Growth curves are representative of three independent tests.

We generated a deletion mutant strain for each *YLJEN* gene (Fig. S2, Table1) and studied their growth in media that each contained one of the following carbon sources: 0.3% glucose, 0.3% acetate, 0.3% butyrate, 0.3% citrate, 0.3% fumarate, 0.3% glutarate, 0.3% DL-lactate, 0.3% malate, 0.3% oxaloacetate, 0.3% pyruvate, and, 0.3% succinate. Surprisingly, we found that *Y. lipolytica* did not grow on glutarate-containing medium (data not shown), suggesting one of three potential scenarios: (1) this yeast does not possess a transporter for this compound, (2) the glutarate concentration was too low to support observable growth, or (3) *Y. lipolytica* is not able to metabolize glutarate. The single-deletion strains grew in the presence of all other carbon sources tested, with the exception of Δ*YLjen1* when fumarate, malate, or succinate are added into the medium (Fig.4A–C and data not shown). Interestingly, this mutant did not grow at all in succinate-based medium, a result which suggests that YLJen1p may be the only, or at least the main, succinate transporter in *Y. lipolytica* (Fig.4C). Moreover, the growth of this strain was strongly reduced in media that contained fumarate or malate as the sole carbon sources (Fig.4A and B), a finding which indicates that YLJen1p is also involved in the transport of these compounds. However, the fact that the strain grew at all demonstrates that other YLJenp proteins are also involved in the transport of fumarate and malate.

### YLJen6p and YLJen1p are the main transporters of malate and fumarate

To determine the proteins involved in the transport of malate and fumarate, several deletion mutant strains were created in which different combinations of Jen proteins were absent (Fig. S2, Table3). We then evaluated their ability to grow on media that contained different carbon sources using the same procedure as for the single-deletion mutants. Interestingly, the quadruple-deletion strains Δ*YLjen*2-5 and Δ*YLjen2,3,5,6* grew as well as the reference strain JMY2900 on all the carbon sources tested (Fig.4D–F and data not shown), a result showing that *YLJEN2*, *YLJEN3*, *YLJEN4*, *YLJEN5*, and *YLJEN6* are not necessary for growth on the substrates tested here. Moreover, mutants in which *YLJEN2*, *YLJEN3*, *YLJEN4*, and/or *YLJEN5* were deleted together with *YLJEN1* exhibited the same reduction in growth on malate- and fumarate-based media as the Δ*YLjen1* mutant (data not shown). This result confirms that *YLJEN2*, *YLJEN3*, *YLJEN4*, and *YLJEN5* are not involved, or are only weakly involved, in the transport of fumarate and malate. This finding was not surprising with regard to *YLJEN3* and *YLJEN4* because they were not expressed even in the wild-type strain, but it was unexpected for *YLJEN2* and *YLJEN5* because both are expressed in the wild-type under the conditions tested here. Nevertheless, the deletion of *YLJEN6* together with *YLJEN1* was sufficient to prevent growth on malate medium and strongly reduce growth on fumarate medium (Fig.4D and E). This result reveals that these two genes serve as the main transporters of malate and fumarate in *Y. lipolytica*. An absence of growth on fumarate medium was observed when, in addition to *YLJEN1* and *YLJEN6*, *YLJEN2,* and *YLJEN5* or *YLJEN3* and *YLJEN5* were deleted (data not shown). This shows that YLJen5p is also able, at least weakly, to transport fumarate. Analysis of the quintuple mutant Δ*YLjen1,2,3,5,6* and sextuple mutant Δ*YLjen1-6* also reveal an absence of growth in media containing fumarate, malate*,* or succinate (Fig.4D–F). Surprisingly, Δ*YLjen1-6* grew as well as JMY2900 in media containing 0.3% acetate, 0.3% butyrate, 0.3% citrate, 0.3% DL-lactate, 0.3% pyruvate, and 0.3% oxaloacetate (Fig. S3). It thus seems that YLJen proteins are not involved in the transport of these substrates, although we cannot exclude the possibility, especially for oxaloacetate, that other transporter(s) also take up these kinds of substrates and therefore compensate for the absence of the six *YLJEN* genes.

### Overexpression of YLJen proteins rescues the transport of fumarate, malate, and succinate in the sextuple-deletion mutant Δ*YLjen1-6*

**Figure 5:**
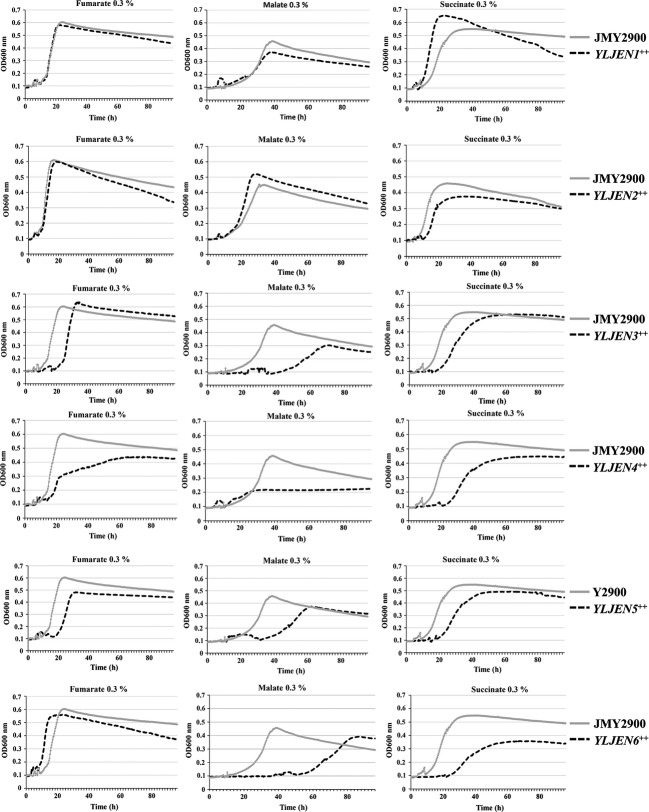
Growth of *YLJEN*-overexpression Δ*YLjen1-6* mutants on media containing 0.3% fumarate, malate, or succinate. Growth curves are representative of three independent tests.

To better understand the specificity of the YLJenp, we analyzed the ability of each protein to rescue substrate-transport functions in the sextuple-deletion mutant Δ*YLjen1-6*. Each gene was expressed under the strong and constitutive promoter p*TEF*. We then evaluated the ability of each constructed strain to grow in media containing fumarate, malate, or succinate (Fig.5). As expected, the overexpression of *YLJEN1* completely restored growth in the three types of media (Fig.5). However, the overexpression of *YLJEN2* also had similar effects (Fig.5), which was surprising given that its deletion had not any observable effect on the growth of *Y. lipolytica* in these media. Unexpectedly, strains that overexpressed *YLJEN5* or *YLJEN3* grew almost as well as the *YLJEN1*-overexpression mutant in media containing fumarate or succinate (Fig.5), whereas, under the same conditions in the JMY2900 wild-type strain, these genes were poorly or not expressed (Fig.3). However, their overexpression only partially rescued the growth of Δ*YLjen1-6* in malate medium (Fig.5), as would be expected from the RT-PCR results (no or weak expression in malate-based medium, Fig.3). Although *YLJEN4* was not expressed in JMY2900, its overexpression partially restored growth in succinate and fumarate media and, to a much lesser degree, also in malate medium (Fig.5). *YLJEN6* restored the growth of Δ*YLjen1-6* completely in fumarate-based medium but only weakly in malate and succinate media (Fig.5). This result was surprising given the RT-PCR results, which had shown that *YLJEN6* was strongly induced in the presence of fumarate and malate but not expressed in succinate medium (Fig.3).

Taken together, these results indicate that (1) YLJen1p and YLJen2p are generalist transporters with no specific substrate affinity; (2) YLJen3p, YLJen4p, and YLJen5p are more specialized for the transport of fumarate and succinate; and (3) YLJen6p displays a high degree of affinity for fumarate. The best transporters, both in terms of transport efficiency and the diversity of substrates transported, are therefore YLJen1p and YLJen2p, which derivate from a common ancestor in the Jen3 phylogenetic tree (Fig.2B). It is possible, then, that their malate-transporting capabilities may derive from a common ancestor. To test this hypothesis, we attempted to investigate Jen protein from *C. hispaniensis,* a species closely related to *Y. lipolytica*, at the base of the *Yarrowia* clade (Kurtzman 2005; Michely et al. 2013). This species successfully grew in glucose-based medium, but was unable to grow in media that contained, alone or in mixture, lactate, malate, succinate, or fumarate (data not shown), suggesting that CHJenp is nonfunctional. We then overexpressed *CHJEN* in the Δ*YLjen1*-*6* mutant, and found that this strain did not grow in media containing lactate, malate, succinate, and/or fumarate (data not shown), a result which lent support to this hypothesis.

In showing that multiple-*YLJEN* genes participate in fumarate transport, our results provide an explanation for the diversity of phenotypes found in the different deletion mutants grown with this substrate. However, it is very curious that, in vivo, the deletion of *YLJEN1* completely inhibited growth in succinate-based medium, because our overexpression analysis revealed that YLJen2p and YLJen5p can also transport this compound (YLJen3p and YLJen4p can also transport succinate but they are not expressed in JMY2900). It is therefore possible that the transport of succinate is highly regulated. The same observation can be made about malate transport, as the deletion of *YLJEN1* with *YLJEN6* prevented growth, even though *YLJEN5* and more specially *YLJEN2* are also able to transport malate. One explanation could be that YLJen1p and/or YLJen6p control expression of the other *YLJEN* genes. However, RT-PCR performed in Δ*YLjen1* and Δ*YLjen1*Δ*YLjen6* deletion mutants revealed that the remaining *JEN* genes had the same expression profile (data not shown) as seen in the wild-type (Fig.3). This result indicates that YLJen1p and YLJen6p do not regulate the expression of the other *YLJEN* genes, at least at a transcriptional level. Posttranslational regulation may exist and could explain our results. It therefore seems that, as has been shown in *S. cerevisiae* (Andrade and Casal 2001; Paiva et al. 2002; Andrade et al. 2005), the regulation of *JEN* genes in *Y. lipolytica* has the potential to be very complex.

### Patterns of carbon-source consumption in YLJEN-overexpression strains confirm substrate specificities of most YLJenp transporters

To better characterize the specificity and transport capacity of each YLJen protein, we used HPLC analysis to analyze the consumption of fumarate, malate, and succinate in the wild-type strain JMY2900, the deletion mutants Δ*YLjen1*, Δ*YLjen6,* Δ*YLjen1,6*, Δ*YLjen1-6*, and the six *YLJEN*-overexpression mutants. Strains were cultivated in YNB medium supplemented with 170 mmol/L of fumarate, malate, succinate, and lactate; lactate was added to enable the survival of strains, such as Δ*YLjen1*-*6*, that are not able to use other carbon sources.

**Figure 6:**
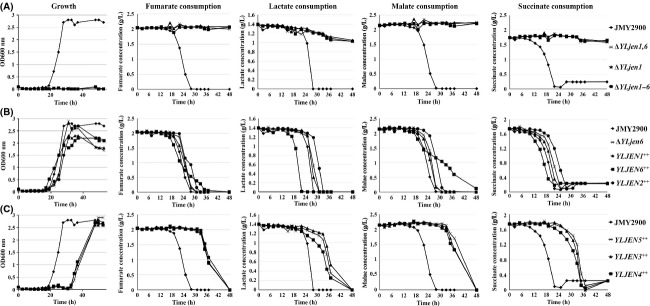
Growth and carbon-source consumption of reference strain JMY2900; *YLJEN1*-, *YLJEN6*-, and *YLJEN1,6*-deletion mutants; and *YLJEN*-overexpression strains. Strains were grown on media which contained fumarate, lactate, malate, or succinate, and three patterns of strain behavior were observed, presented here in A, B, and C, respectively. Growth and consumption curves are representative of three independent tests.

Interestingly, three general patterns of strain behavior were observed. In the first, strains did not grow and did not consume fumarate, malate, and succinate; this group included Δ*YLjen1-6*, Δ*YLjen1*, and Δ*YLjen1,6* (Fig.6A). However, the lactate concentration in the medium decreased slowly, at a rate of about 0.008 g L^−1^ h^−1^. This rate was 26-fold lower that that observed for the reference strain JMY2900 (0.21 g L^−1^ h^−1^), suggesting that these strains consumed lactate solely for the purpose of cell maintenance. Based on the results of our previous experiments, we expected that Δ*YLjen1,6* and Δ*YLjen1-6* would not grow in this medium; however, it was surprising to see that the Δ*YLjen1* mutant did not grow either. One potential explanation for this could be that the culture conditions in this experiment were different from those used to generate the results presented in Figure4. Indeed, our initial growth experiments were conducted with a Biotek apparatus using media that contained only one carbon source, while the consumption tests were performed in flasks using a medium that contained four different carbon sources. Moreover, we note that the lag phase for JMY2900 in the growth experiments lasted around 10 h (Fig.4) compared to 20 h in the consumption experiments (Fig.6), an observation which suggests that cells may have been more stressed in the latter conditions.

The second pattern of strain behavior involved growth that began at approximately 20 h of culture and substrate consumption that was as efficient as or better than that found in the JMY2900 wild-type, at 86–129% of the WT rate of consumption. This group was composed of Δ*YLjen6* and Δ*YLjen1*-*6* strains in which *YLJEN1* or *YLJEN2* were overexpressed (Fig.6B and C4). Finally, there was a third identifiable pattern: in this group, strains began to grow after approximately 30 h in culture and consumption rates of at least one substrate was lower than those found in the case of JMY2900. This group contained Δ*YLjen1*-*6* strains that overexpressed *YLJEN5* (consumption rate of malate reduced 2.2-fold compared to WT), *YLJEN3* (consumption rate of malate and lactate reduced 1.4- and 1.3-fold, respectively), and *YLJEN4* (consumption rate of malate and lactate reduced 1.5- and 1.75-fold, respectively) (Fig.6C and D4). In general, the results of the consumption tests were consistent with the growth curves shown in Figures4 and 5 in showing that members of group 2 (i.e., strains that overexpressed *YLJEN1* or *YLJEN2*) grew similarly to JMY2900 in the different substrates while members of group 3 (i.e., strains that overexpressed *YLJEN3, YLJEN4,* or *YLJEN5*) grew more slowly in malate.

**Table 4:** Substrate consumption rates of the different strains

	Fumarate		Malate		Succinate		Lactate	
	Rate (g L^−1^ h^−1^)	*R* ^2^	Rate (g L^−1^ h^−1^)	*R* ^2^	Rate (g L^−1^ h^−1^)	*R* ^2^	Rate (g L^−1^ h^−1^)	*R* ^2^
Reference strain JMY2900	0.22 (1)	0.98	0.24 (1)	0.96	0.19 (1)	0.97	0.21 (1)	0.91
Δ*YLjen6*	0.28 (1.2)	0.99	0.29 (1.2)	0.95	0.18 (0.95)	0.99	0.20 (0.94)	0.99
*YLJEN1*^*++*^	0.24 (1.1)	0.99	0.27 (1.13)	0.99	0.21 (1.1)	0.97	0.20 (1.1)	0.92
*YLJEN2*^*++*^	0.19 (0.88)	0.96	0.29 (1.2)	0.99	0.17 (0.86)	0.99	0.24 (0.86)	0.99
*YLJEN3*^*++*^	0.26 (1.2)	0.99	0.18 (0.74)	1	0.29 (1.52)	0.94	0.16 (0.77)	0.98
*YLJEN4*^*++*^	0.24 (1.1)	0.99	0.16 (0.65)	0.96	0.23 (1.2)	0.97	0.12 (0.57)	0.96
*YLJEN5*^*++*^	0.22 (1)	1	0.10 (0.44)	0.97	0.27 (1.4)	0.94	0.18 (0.85)	0.99
*YLJEN6*^*++*^	0.16 (0.74)* 0.057 (0.26)**	0.990.99	0.12 (0.49)* 0045 (0.19)**	10.99	0.2 (1.05)	1	0.25 (1.14)	0.96

Values in parenthesis indicate ratio of rate of mutant to rate of reference strain, that is rate of substrate consumption before (*) and after (**) 22 h of growth. The results represent the mean values of at least two independent experiments and SD did not exceed 7%. *R*^2^ corresponds to the coefficient of linear correlation.

Interestingly, we observed a pattern of growth in the *YLJEN6*-overexpression strain of Δ*YLjen1*-*6* which displayed characteristics of both groups 2 and 3. It began to grow after approximately 20 h in culture (more slowly than the other strains in group 2) and it consumed fumarate and malate more slowly as well (consumption rate decreased 1.35- and 2-fold, respectively, until 22 h of growth; Fig.6B and C4). Moreover, this strain showed some substrate specificity. Indeed, at 22 h, a point at which the supply of succinate and lactate in the medium had been exhausted, the consumption rate of malate and fumarate decreased drastically and simultaneously, 2.6- and 2.9-fold respectively, but was not followed by an immediate decrease in growth rate. The decrease in growth rate that happened at 28 h of culture could be caused by this change in substrate consumption (Fig.6 and Table4). In our gene-rescue experiments, we found that overexpression of *YLJEN6* did not completely restore growth in succinate- and malate-based media (Fig.5), a finding which suggests that YLJen6p is not able to efficiently transport these compounds. The strong consumption rate of succinate in the *YLJEN6*-overexpression strain, then, is surprising, and could be explained by the presence of lactate, which may potentially be co-transported with succinate, and thus increase the rate of uptake. Similarly, the observation that the rate of fumarate consumption in the *YLJEN6*-overexpression strain was much weaker here compared to the efficient utilization of fumarate by this strain in the gene-rescue experiment (Fig.5) could be due to transport inhibition by malate.

### Lactate and fumarate facilitate succinate consumption in the YLJEN6-overexpression strain

**Figure 7:**
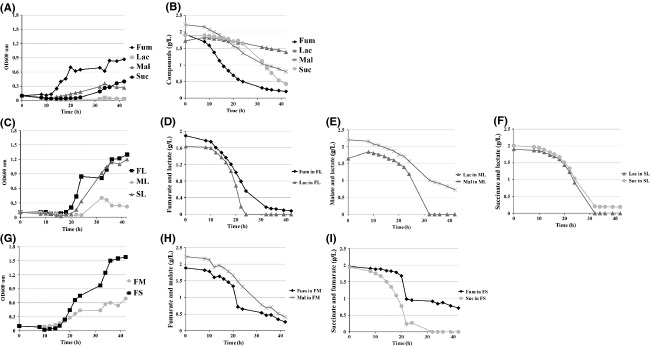
Growth and carbon-source consumption of the *YLJEN6*-overexpression strain when grown on media which contained, alone or in mixture, fumarate, lactate, malate, and/or succinate. (A) Growth in media containing fumarate (Fum), lactate (Lac), malate (Mal), or succinate (Suc). (B) Substrate consumption in media containing fumarate (Fum), lactate (Lac), malate (Mal), or succinate (Suc). (C) Growth in media containing lactate and fumarate (FL), lactate and malate (ML), or lactate and succinate (SL). (D) Substrate consumption in media containing lactate and fumarate. (E) Substrate consumption in media containing lactate and malate. (F) Substrate consumption in media containing lactate and succinate. (G) Growth in media containing fumarate and malate (FM) or fumarate and succinate (FS). (H) Substrate consumption in media containing fumarate and malate (I) Substrate consumption in media containing fumarate and succinate.

To test these hypotheses, we cultivated *YLJEN6*-overexpression Δ*YLjen1*-*6* mutants in various media, containing several combinations of mono- and di-acids (fumarate only; lactate only; malate only; succinate only; fumarate and lactate; malate and lactate; succinate and lactate; fumarate and malate; fumarate and succinate). Growth and substrate consumption were quantified by spectroscopy and HPLC, respectively (Fig.7). Growth in media which contained only fumarate, malate, or succinate was similar to that shown in Figure5; however, this strain did not grow in lactate-based medium (Fig.7A). This was explained by the too low concentration of lactate in this experiment (170 mmol/L vs. 340 mmol/L in the growth experiment above; data not shown). Because of this lack of growth, lactate was consumed very slowly (rate = 0.013 g L^−1^ h^−1^; *R* = 0.99; Fig.7B), similarly to what had been observed in the previous experiment for the Δ*YLjen1*-*6* strain (Fig.6), and indicating that it was only used for cell maintenance and survival. As expected, the patterns of consumption of fumarate, malate, and succinate reflected those found in the growth curves: fumarate was consumed rapidly (rate = 0.11 g L^−1^ h^−1^; *R* = 0.99), while malate and succinate were consumed slowly (at respective rates of 0.04 g L^−1^ h^−1^; *R* = 0.98 and 0.07 g L^−1^ h^−1^; *R* = 0.99; Fig.7B).

Interestingly, the addition of lactate to media containing fumarate or succinate improved both the growth rate and the amount of growth achieved by the *YLJEN6*-overexpression strain (Fig.7C); however, its addition to malate medium had no effect (Fig.7C). In these media, lactate was consumed more rapidly than either fumarate (rate = 0.21 g L^−1^ lactate h^−1^; *R* = 0.98 vs. rate = 0.1 g L^−1^ fumarate h^−1^; *R* = 0.99) or malate (rate = 0.14 g lactate L^−1^ h^−1^; *R* = 0.99 vs. rate = 0.05 g malate L^−1^ h^−1^; *R* = 0.98), an observation which suggests that the transport of lactate is not coupled with that of either fumarate or malate (Fig.7D and E). Conversely, the consumption curves of succinate and lactate overlapped (both at a rate of around 0.11 g L^−1^ h^−1^ with *R* = 0.99), providing evidence that they are co-transported (Fig.7F) and supporting our previous hypothesis.

The combination of malate and fumarate in the medium resulted in growth that was weaker than that observed in fumarate-only medium (Fig.7G). This was likely the result of low malate and fumarate consumption rates (rate = 0.06 g.L^−1^ h^−1^; *R* = 0.98 and rate = 0.05 g L^−1^ h^−1^; *R* = 0.98, respectively; Fig.7H). Interestingly, the consumption curves of these two substrates were quite similar. However, in this mixture, the fumarate consumption rate was twofold weaker that than found in the fumarate-only medium (rate = 0.11 g L^−1^ h^−1^). Instead, malate consumption was quite similar to that observed in other malate-containing media. Together, these results suggest that malate somehow inhibited fumarate consumption. We observed better growth on the fumarate and succinate medium. Under these conditions, fumarate was consumed 15-fold more slowly than succinate (with the exception of the period between 20 and 22 h, the consumption rate of fumarate was around 0.01 g L^−1^ h^−1^; *R* = 0.90 vs. 0.16 g L^−1^ h^−1^; *R* = 0.96 for succinate) and after 42 h of culture, there was still approximately 0.6 g L^−1^ fumarate remaining in the medium (Fig.7I). Additionally, the rate of succinate consumption was much higher in this medium than in the succinate-only medium. It seems, therefore, that the presence of fumarate somehow promotes succinate uptake, while succinate inhibits the transport of fumarate.

By evaluating the consumption profiles of fumarate, lactate, malate, and succinate both individually and when mixed together, we can observe that the consumption profiles presented in Figure6 are influenced by the competitive or facilitative roles of the different carbon sources with regard to each other. Although YLJen6p displays some preference for fumarate when only one substrate is present in the medium, its affinity for succinate changes strongly in the presence of lactate or fumarate, compounds which appear to be effectors of succinate uptake.

## Conclusions

Lodi et al. (2007) showed that the Jen proteins in *Y. lipolytica* form a distinct group comparable to those found in *S. cerevisiae*, *K. lactis*, *C. albicans*, and other Hemiascomycetes and Euascomycetes. They assigned *YLJEN2*, *YLJEN3*, *YLJEN4,* and *YLJEN6* to the Jen2 cluster and *YLJEN5* and *YLJEN1* to the Jen1 cluster, and furthermore identified YLJen1p and YLJen5p as “preJen1p” proteins (Lodi et al. 2007). However, through the use of a comparative genomics approach involving species closely related to *Y. lipolytica,* we found that this protein family has a dynamic evolutionary history dating from the emergence of the *Yarrowia* clade and represents a distinct subfamily of Jen proteins, Jen3p. In analyses of single-*YLJEN* and multiple-*YLJEN*-deletion mutants, we observed that growth was unaffected in acetate-, butyrate-, citrate-, DL-lactate-, oxaloacetate-, and pyruvate-based media, suggesting that either YLJen proteins are not involved in the transport of these substrates or there are other transporters that can compensate for their absence. In general, we found that the substrate specificities of the YLJen proteins are similar to members of the Jen2 subfamily, but that the different YLJenp vary a great deal in their expression level and transport targets. Indeed, it seems that YLJen1p is the main, most efficient transporter, followed by YLJen6p, although this will require specific transport essay to determine the kinetic parameters. Interestingly, we found a strong correlation between phylogeny, expression level, and transport targets. For example, YLJen1p and YlJen2p are derived from a common ancestor, transport the same substrates when they are overexpressed (fumarate, malate, and succinate), and demonstrate similar expression patterns, even if *YLJEN1* is up-regulated under more conditions than *YLJEN2* is. We were unable to determine if the YLJenp can transport pyruvate or lactate. However, some *YLJEN* genes, for example, *YLJEN6*, are upregulated by lactate. YLJen6p is also unusual in that exposure to lactate, but also to fumarate, increased succinate uptake in a mutant strain in which YLJen6p was the only functioning Jen protein, an observation which could explain the divergent results we obtained in experiments that involved *YLJEN6* (Figs.5, 6, or 7).

Since *Y. lipolytica* is a an important organism in the biotechnology sector for its ability to produce both lipids (Beopoulos et al. 2011) as well as also other compounds such as succinate (Kamzolova et al. 2009, 2014; Yuzbashev et al. 2010), more investigations on succinate production with a strain devoid of the six *YLJEN* genes could be a promising avenue for future research since the absence of succinate consumption by the mutant strain could increase its production.

## Supporting Information

**Table S1.** Jen proteins found in the Hemiascomycetes.

**Figure S1.** TMHMM profiles of different Jen proteins.

**Figure S2.** Strain construction of the mutants used in this study.

**Figure S3.** Growth of Δ*YLjen1-6* and JMY2900 in media which contained 0.3% acetate, butyrate, citrate, lactate, pyruvate, or oxaloacetate. Δ*YLjen1-6* and JMY2900 growth curves are represented in gray and black, respectively. Growth curves are representative of three independent tests.
