# Supplementary figures and images for: The evolution of Jen3 proteins and their role in dicarboxylic acid transport in Yarrowia
Source: Microbiologyopen. 2014 Dec 16;4(1):100–20. doi: 10.1002/mbo3.225 (PMC4335979; doi:10.1002/mbo3.225)

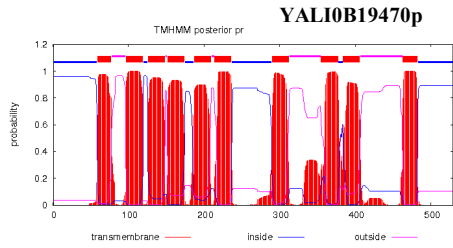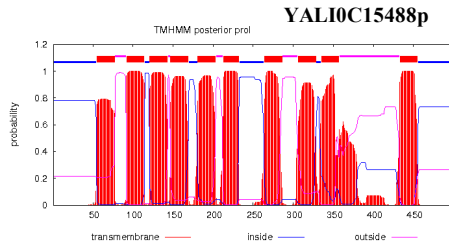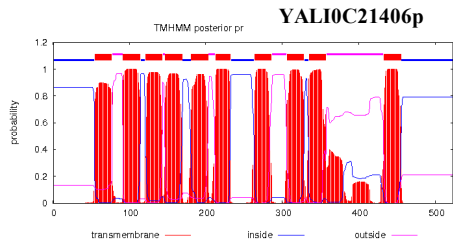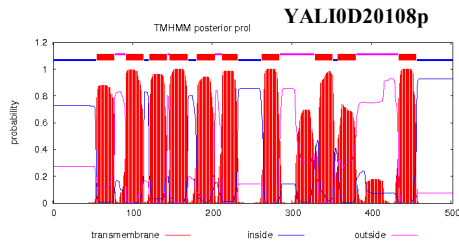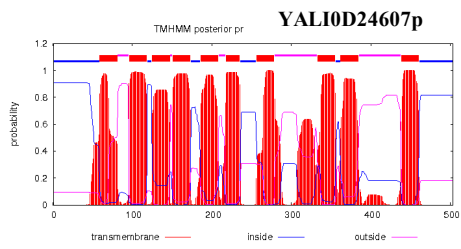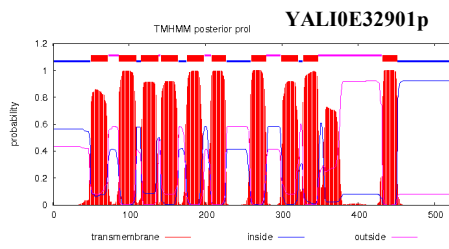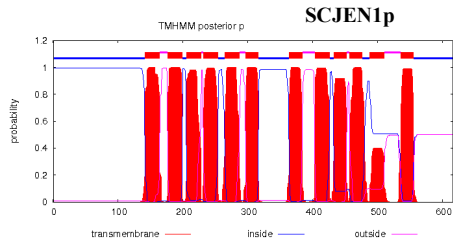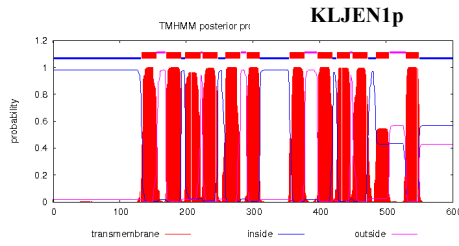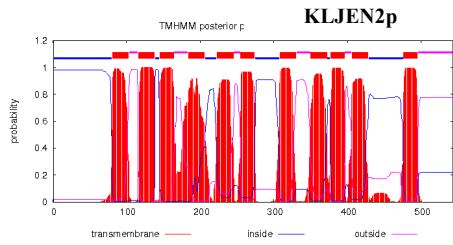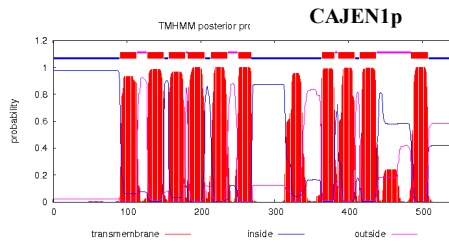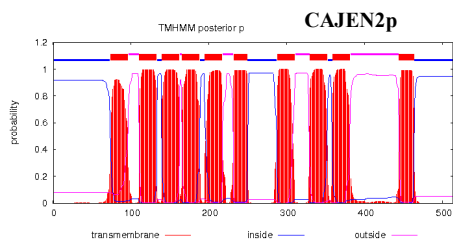

Supplement: Supplementary file 2 [file mbo30004-0100-sd2.pdf]

# P01d

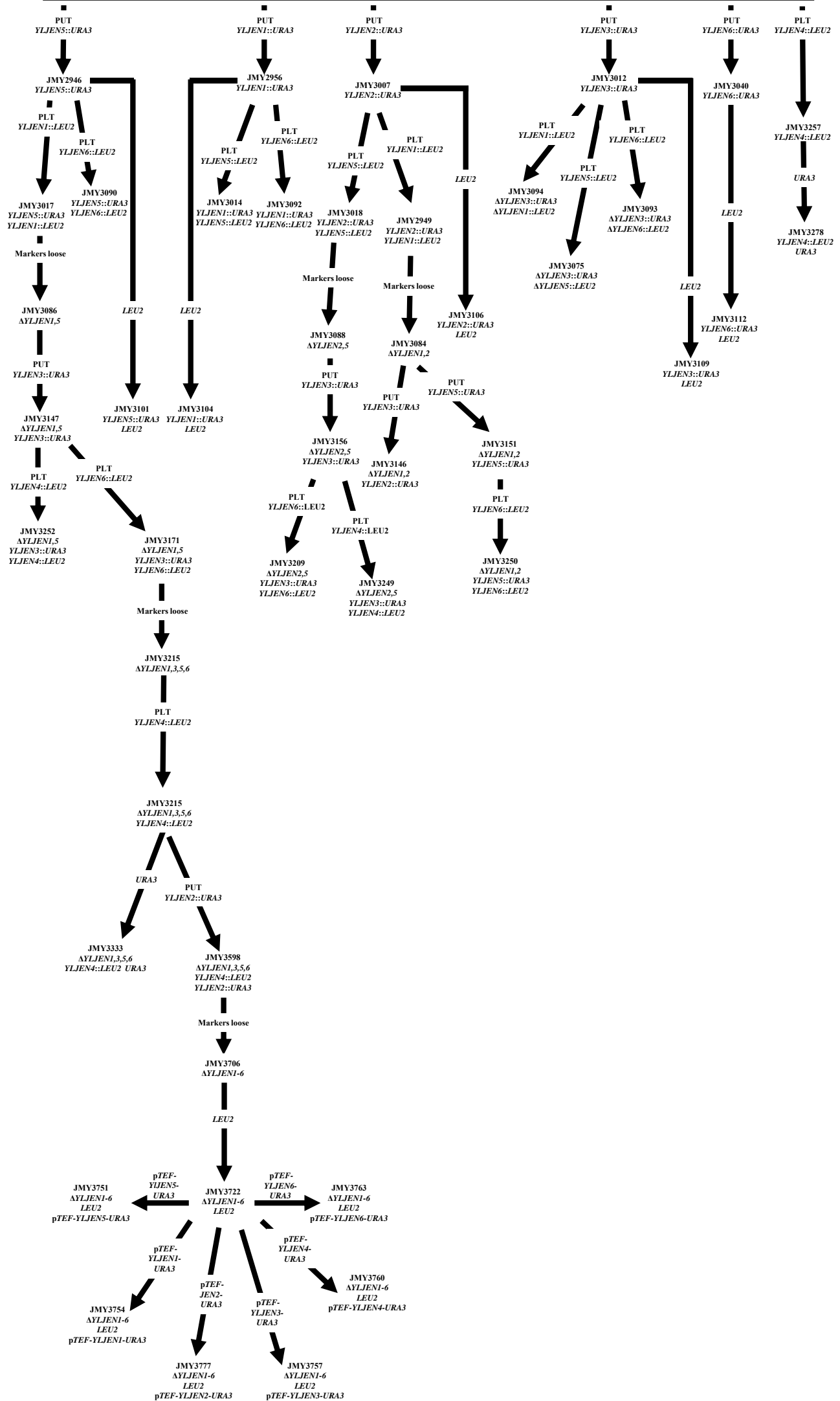

Supplement: Supplementary file 3 [file mbo30004-0100-sd3.pdf]
