# Supplementary material for: The evolution of Jen3 proteins and their role in dicarboxylic acid transport in Yarrowia
Source: Microbiologyopen. 2014 Dec 16;4(1):100–20. doi: 10.1002/mbo3.225 (PMC4335979; doi:10.1002/mbo3.225)
Supplement: Supplementary file 4 [file mbo30004-0100-sd4.pdf]

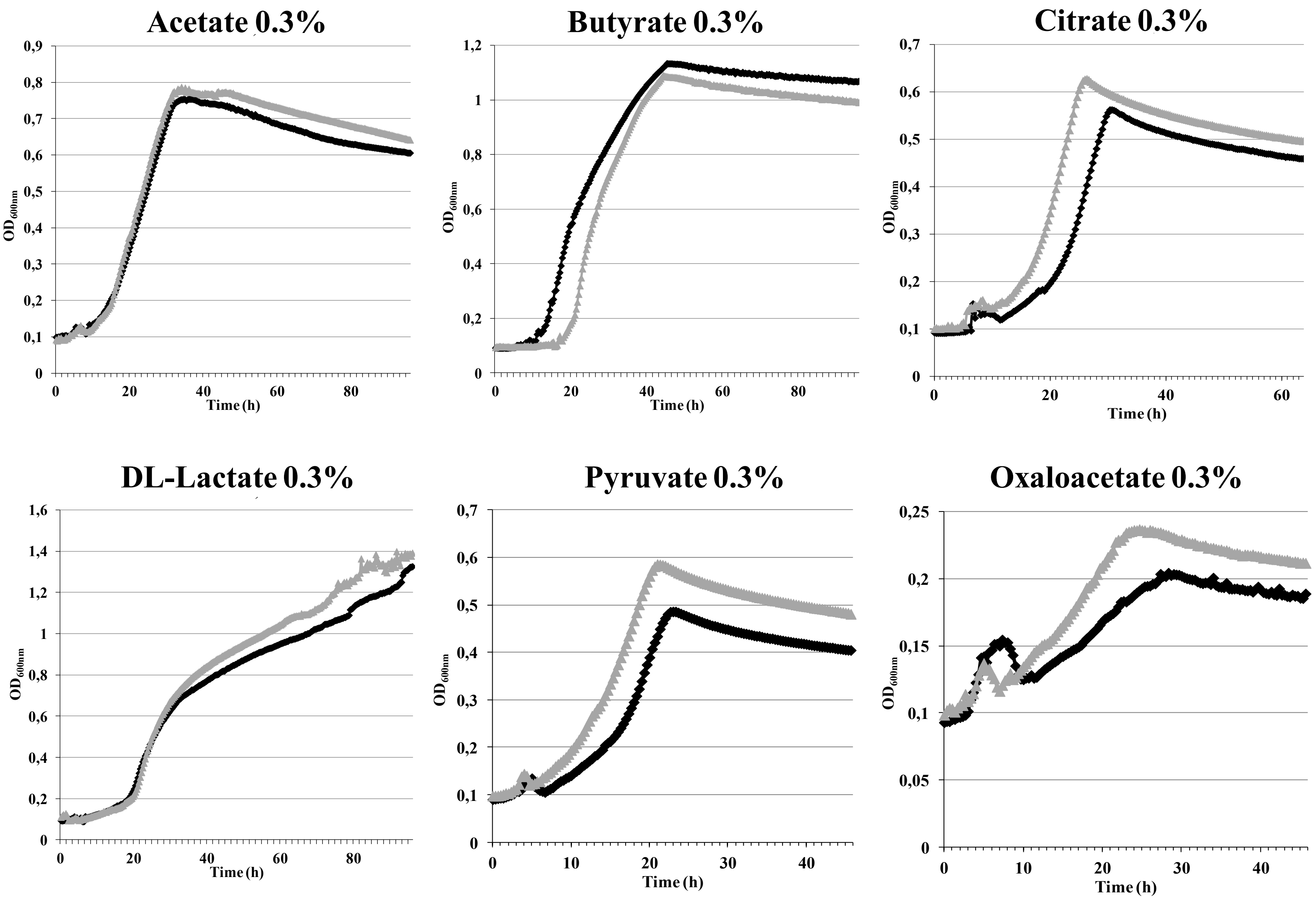

Acetate 0.3%
Butyrate 0.3%
Citrate 0.3%
DL-Lactate 0.3%
Pyruvate 0.3%
Oxaloacetate 0.3%
OD_{600nm}
Time (h)

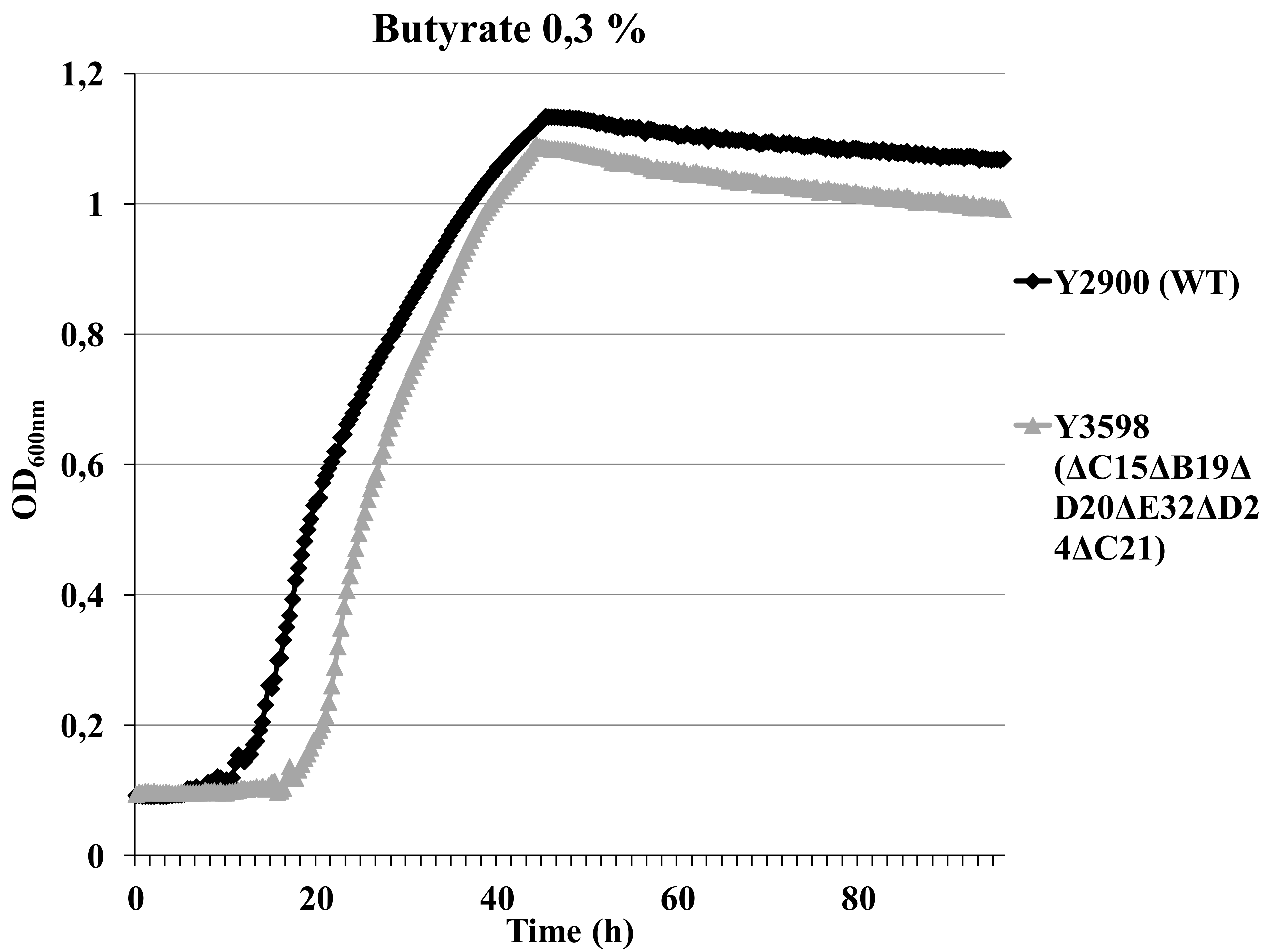

Butyrate 0,3 %
1,2
1
0,8
0,6
0,4
0,2
0
OD600nm
0
20
40
60
80
Time (h)
Y2900 (WT)
Y3598 (ΔC15ΔB19ΔD20ΔE32ΔD24ΔC21)

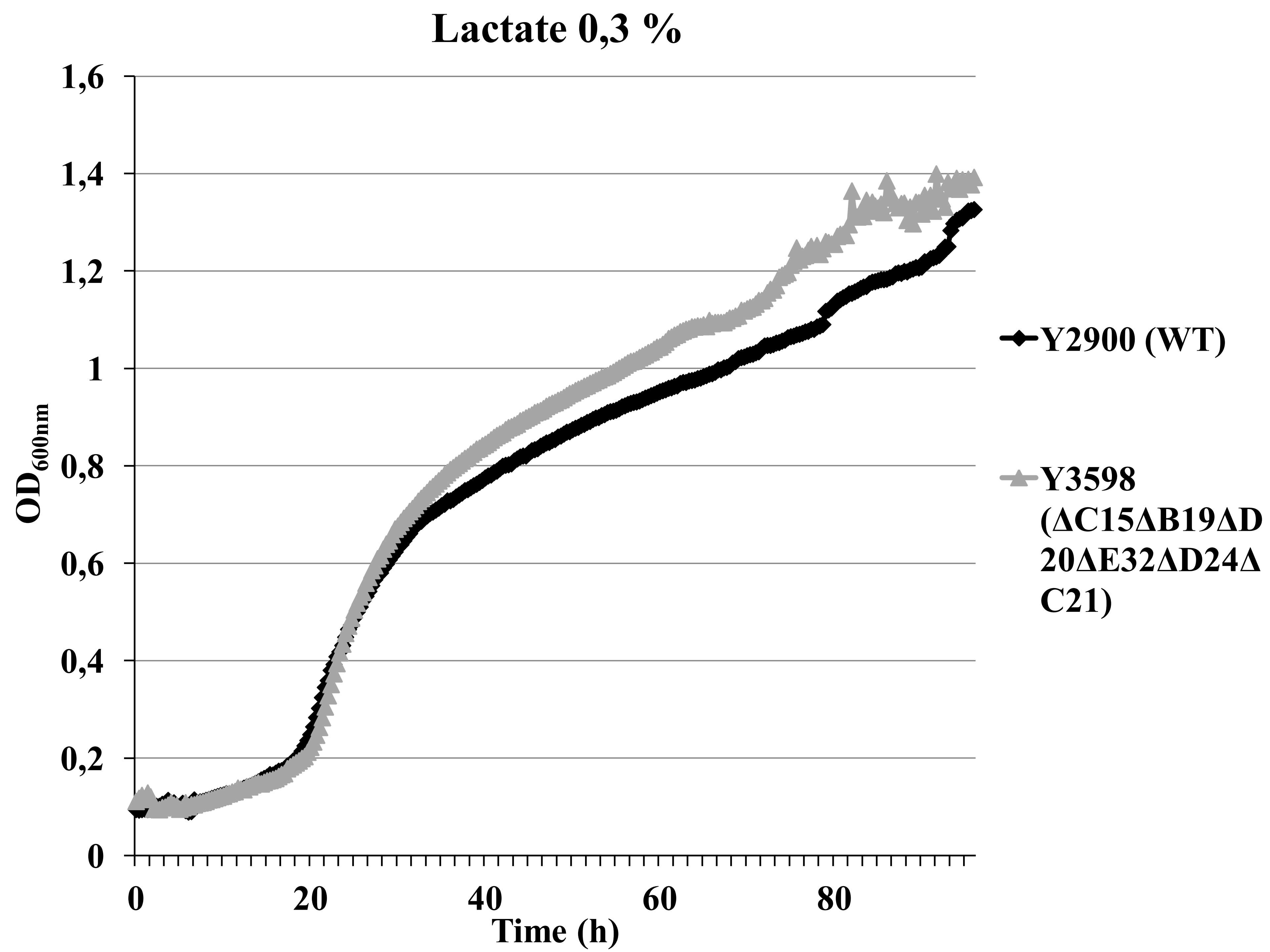

Lactate 0,3 %
OD600nm
1,6
1,4
1,2
1
0,8
0,6
0,4
0,2
0
0
20
40
60
80
Time (h)
Y2900 (WT)
Y3598 (ΔC15ΔB19ΔD20ΔE32ΔD24ΔC21)

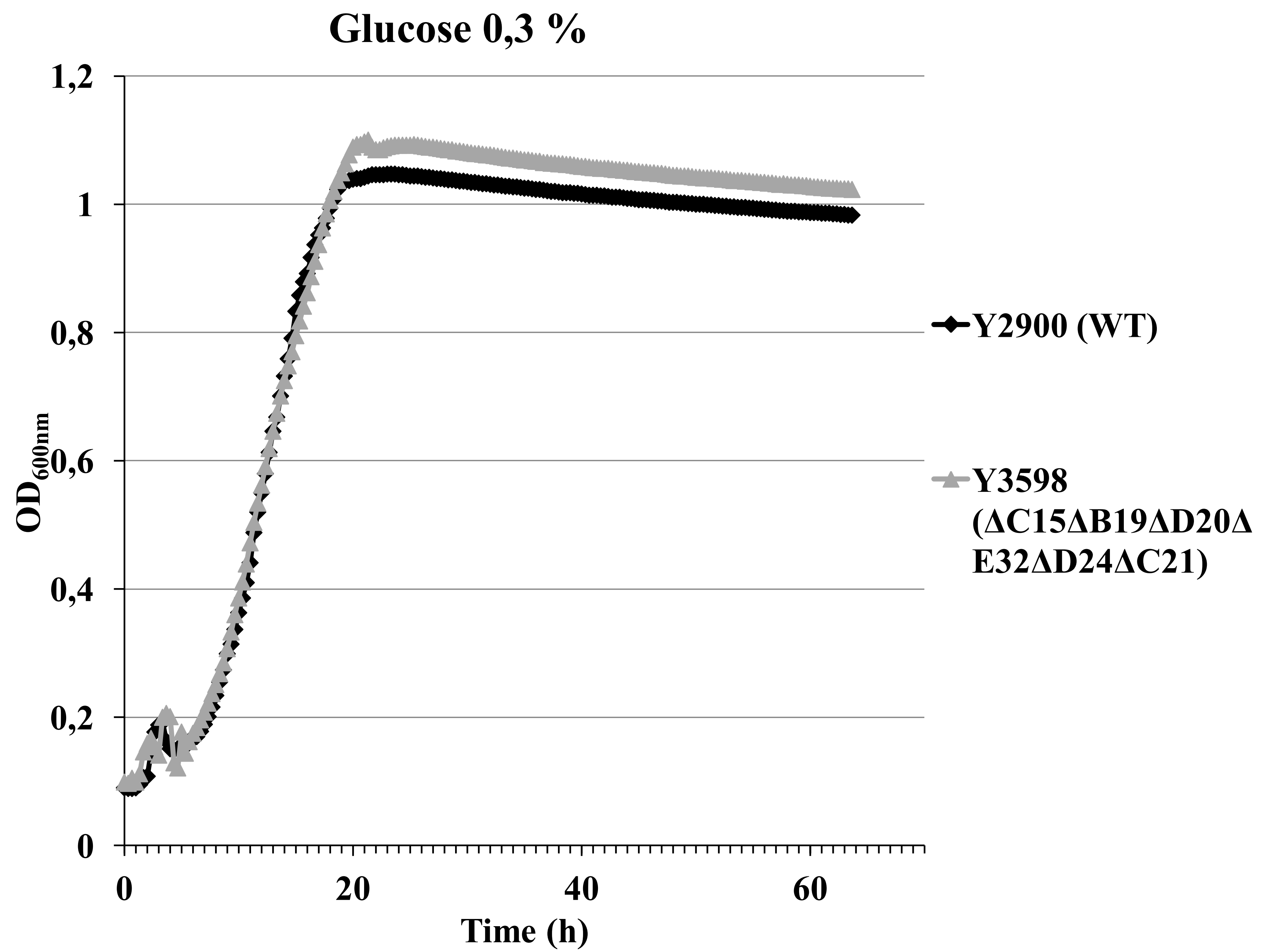

Glucose 0,3 %
1,2
1
0,8
0,6
0,4
0,2
0
0
20
40
60
Time (h)
OD600nm
Y2900 (WT)
Y3598 (ΔC15ΔB19ΔD20ΔE32ΔD24ΔC21)

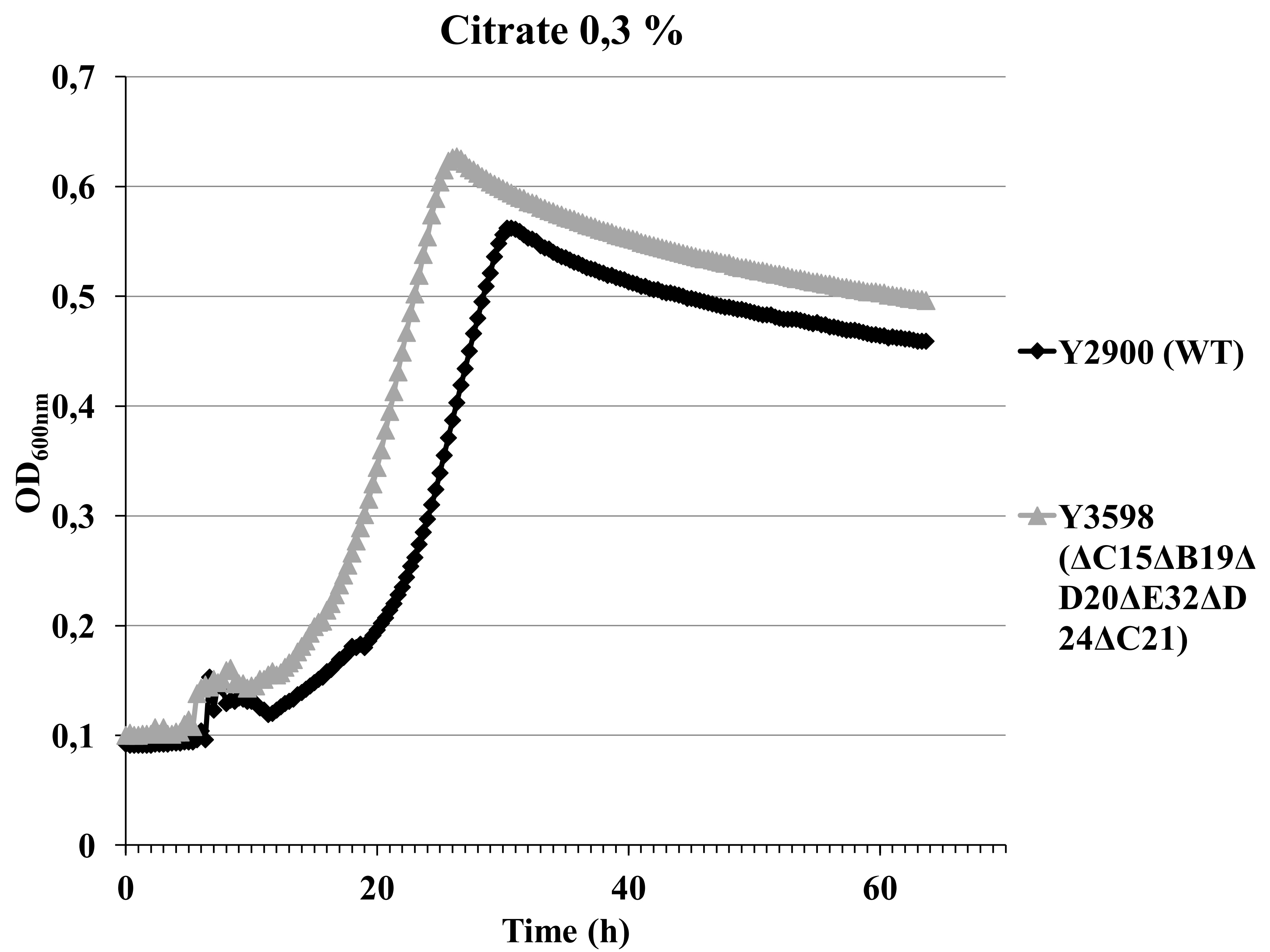
Citrate 0,3 %
0,7
0,6
0,5
0,4
0,3
0,2
0,1
0
OD_{600nm}
0
20
40
60
Time (h)
Y2900 (WT)
Y3598 (ΔC15ΔB19ΔD20ΔE32ΔD24ΔC21)

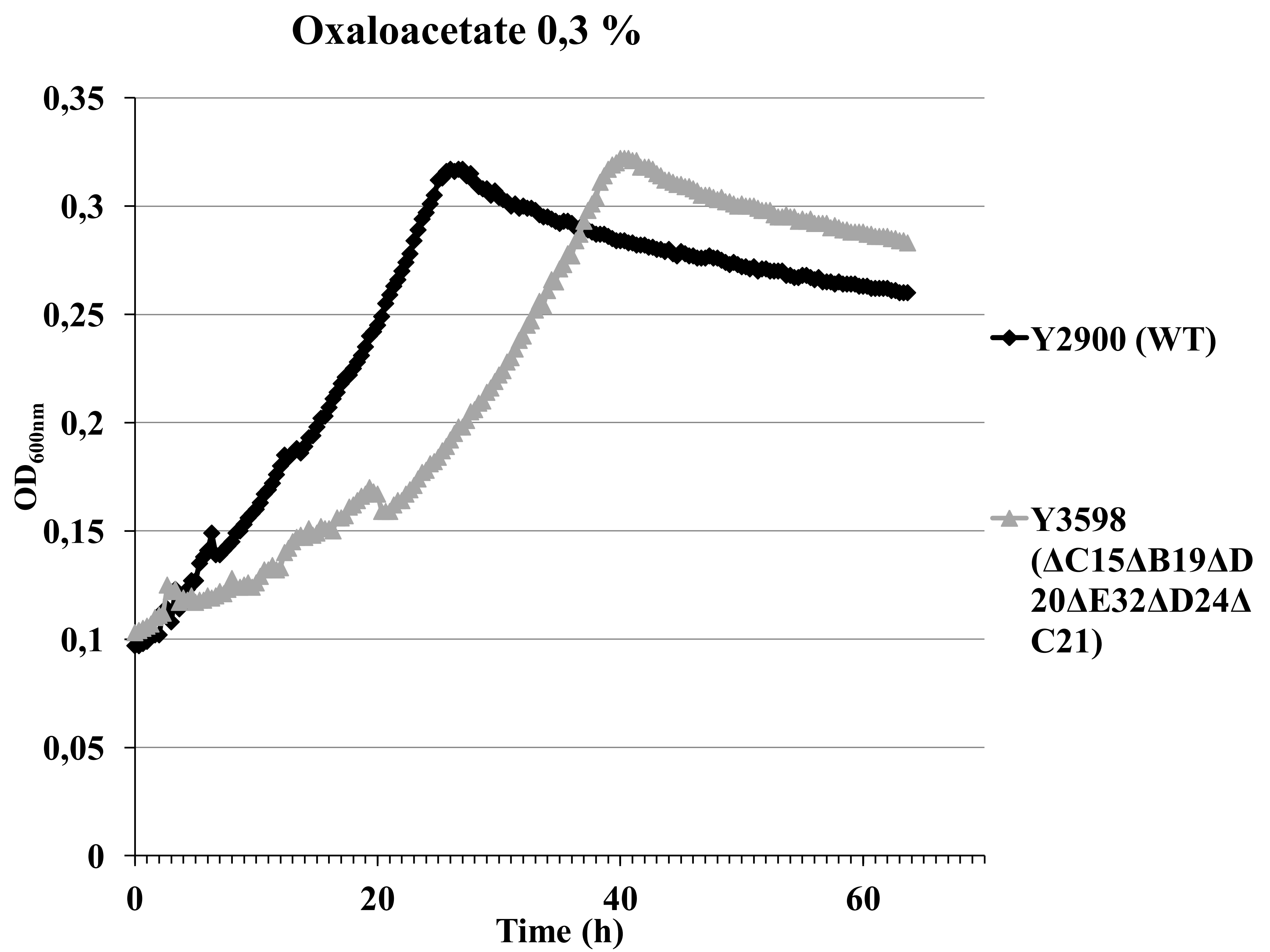

Oxaloacetate 0,3 %
0,35
0,3
0,25
0,2
0,15
0,1
0,05
0
OD600nm
0
20
40
60
Time (h)
Y2900 (WT)
Y3598 (ΔC15ΔB19ΔD20ΔE32ΔD24ΔC21)
